# Multi-omic dissection of the cold resistance traits of white water lily

**DOI:** 10.1093/hr/uhae093

**Published:** 2024-02-17

**Authors:** Penghe Qiu, Tong Liu, Yingchun Xu, Chunxiu Ye, Ran Zhang, Yanjie Wang, Qijiang Jin

**Affiliations:** Key Laboratory of Landscaping, Ministry of Agriculture and Rural Affairs, Key Laboratory of State Forestry and Grassland Administration on Biology of Ornamental Plants in East China, College of Horticulture, Nanjing Agricultural University, Nanjing 210095, China; Key Laboratory of Landscaping, Ministry of Agriculture and Rural Affairs, Key Laboratory of State Forestry and Grassland Administration on Biology of Ornamental Plants in East China, College of Horticulture, Nanjing Agricultural University, Nanjing 210095, China; Key Laboratory of Landscaping, Ministry of Agriculture and Rural Affairs, Key Laboratory of State Forestry and Grassland Administration on Biology of Ornamental Plants in East China, College of Horticulture, Nanjing Agricultural University, Nanjing 210095, China; College of Forestry and Landscape Architecture, Xinjiang Agricultural University, Urumqi, China; Key Laboratory of Landscaping, Ministry of Agriculture and Rural Affairs, Key Laboratory of State Forestry and Grassland Administration on Biology of Ornamental Plants in East China, College of Horticulture, Nanjing Agricultural University, Nanjing 210095, China; Key Laboratory of Landscaping, Ministry of Agriculture and Rural Affairs, Key Laboratory of State Forestry and Grassland Administration on Biology of Ornamental Plants in East China, College of Horticulture, Nanjing Agricultural University, Nanjing 210095, China; Key Laboratory of Landscaping, Ministry of Agriculture and Rural Affairs, Key Laboratory of State Forestry and Grassland Administration on Biology of Ornamental Plants in East China, College of Horticulture, Nanjing Agricultural University, Nanjing 210095, China

## Abstract

The white water lily (*Nymphaea candida*), exemplifying nature’s resilience, thrives in the high-altitude terrains of Xinjiang, China, serving as an ideal model for investigating cold adaptation mechanisms in aquatic plants. This study meticulously elucidates the complex cold adaptation mechanisms of the white water lily through a comprehensive and integrated methodological approach. We discovered that the water lily undergoes ecodormancy in winter, retaining high cellular viability and growth potential. During overwintering, the white water lily demonstrates effective resource reallocation, a process facilitated by morphological adjustments, thereby strengthening its resistance to cold temperatures. This enhancement is achieved particularly through the compartmentalization of large vacuoles, the accumulation of osmoregulatory substances, and an increased antioxidant capacity. We established the first exhaustive full-length transcriptome for the white water lily. A subsequent comprehensive analysis of the transcriptome, phytohormones, and metabolome uncovered a multifaceted regulatory network orchestrating cold adaptation. Our research spotlights phytohormone signaling, amino acid metabolism, and circadian rhythms as key elements in the water lily’s defense against cold. The results emphasize the critical role of nitrogen metabolism, especially amino acid-related pathways, during cold stress. Metabolite profiling revealed the importance of compounds like myo-inositol and L-proline in enhancing cold tolerance. Remarkably, our study demonstrates that the white water lily notably diminishes the utilization of unsaturated fatty acids in its temperature regulation strategies. In conclusion, this research substantially enriches our understanding of the white water lily’s intricate cold adaptation mechanisms, offering new perspectives on the adaptive strategies of aquatic plants and potential applications in agricultural advancement.

## Introduction

The ability of plants to adapt to diverse environmental conditions demonstrates their evolutionary resilience. One of the more pronounced environmental constraints is low temperature, which can significantly hinder plant growth and dictate their geographical distributions [1]. In response to such challenges, plants have evolved myriad physiological and biochemical adaptations. Central to these adaptations is the plant’s response to cold stress, which triggers beneficial physiological and biochemical changes.

These adaptations range from osmotic adjustments to modulations in antioxidant enzyme activities, ensuring survival in severe environmental conditions [2]. Concurrently, cold stress induces the augmentation of key metabolites, such as soluble sugar, proline, glycine betaine, and soluble protein, that act as cellular protective agents [3]. This protective response is further accentuated by the increase in antioxidant enzymes, mediating a balance between the production and removal of reactive oxygen species (ROS) induced by cold [4].

Another pivotal adaptive strategy involves the induction of growth cessation and dormancy, particularly evident in perennial herbs [5]. These plants frequently develop storage organs like rhizomes and bulbs to counteract cold stress. Intriguingly, phytohormones emerge as central players in these processes, governing the onset of plant dormancy and modulating various growth stages [6].

The cellular architecture also exhibits remarkable plasticity in response to cold stress. Plants exhibiting robust cold resistance often maintain the integrity of their organelles and membrane structures under such stressors. Conversely, those with weaker cold resistance witness degradation of their cellular structures, suggesting a correlation between cellular stability and cold tolerance [7].

Recent advancements in the realms of transcriptomics and metabolomics have shed light on the molecular underpinnings of plant cold stress responses. Currently, multiple defense signaling pathways are known to directly influence plant responses to cold stress [8]. Based on whether the response and signaling are dependent on DRE-binding factor 1/CRT binding factor (DREB1/CBF), the cold defense signaling pathways can be classified as CBF-dependent or CBF-independent [9]. *CBF* genes are rapidly induced by non-freezing low temperatures, activating the expression of *COR* genes, which is followed by the accumulation of cryoprotectant substances, such as osmotic regulation substances and anti-freeze proteins [10]. Although *CBF* genes play important roles during cold acclimation in plants, less than 20% of *COR* genes are regulated by *CBF* genes. Several CBF-independent transcription factors (TFs) such as Cold-Responsive Zinc Finger 1 (CZF1), Zinc Finger of the *Arabidopsis thaliana* 12 (ZAT12), Heat Stress Transcription Factor C1 (HSFC1) and Elongated hypocotyl 5 (HY5) have been identified to modulate *COR* expression. HY5 is a basic Leucine Zipper (bZIP) transcription factor, which is involved in plant light signaling. HY5 is strongly induced at low temperatures, thereby promoting *COR* gene expression and cold acclimation [11].

Water lilies (*Nymphaea* spp.), belonging to the family *Nymphaeaceae*, are perennial aquatic herbaceous plants renowned for their diverse flower colors, elegant postures, and far-reaching fragrance. These attributes have established them as significant ornamental horticultural plants and crucial basal angiosperms, pivotal to the study of the origins and evolution of flowering plants [12, 13]. Recent advancements in genomics have significantly propelled the related research of water lilies. The genome sequencing of *Nymphaea colorata* [14], *Nymphaea thermarum* [15], and *Euryale ferox* [16] marks a substantial stride in understanding their genetics. These genomic insights are instrumental for in-depth studies of water lilies, revealing ornamental traits, and environmental adaptability, thereby accelerating molecular breeding in these species.

Water lilies can be classified into two distinct categories based on their ecological habits and geographical distribution: tropical and hardy water lilies. While tropical waterlilies boast a superior ornamental value, their inability to survive winters in non-tropical regions limits their economic potential. Current research into the low-temperature responses of water lilies, though in its early stages, is beginning to make significant strides. One pioneering study in this field is the transgenic approach developed by Yu *et al.* [17], which established, for the first time, a transgenic pipeline using the pollen-tube pathway in water lilies. The approach was then utilized to enhance the cold tolerance of water lilies. Recently, the studies by Khan *et al.* [18] and Ma *et al.* [19] have begun to unravel the molecular mechanisms underlying the cold stress response in water lilies. However, these studies represent only the initial steps in a much-needed comprehensive exploration. Detailed investigations into morphological adaptations, metabolic alterations, and the cold signal transduction network are imperative to fully understand the cold adaptation strategies employed by these plants.

In this context, the white water lily (*Nymphaea candida*), flourishing in the alpine climates of Xinjiang, China, presents a special case. Its distinct ecological niche renders it an invaluable subject for exploring cold adaptation mechanisms in aquatic plants. Despite this, the mechanisms underpinning its ability to withstand extreme low temperatures remain enigmatic. This study ventures into the uncharted territory of the white water lily’s cold adaptation mechanisms, aiming to glean insights that could inform the cold resistance breeding of tropical waterlilies. Moreover, these findings have the potential to be extrapolated to enhance cold resistance in other plant species, particularly economically significant crops.

## Results

### The morphological and developmental changes of white water lily

**Figure 1:**
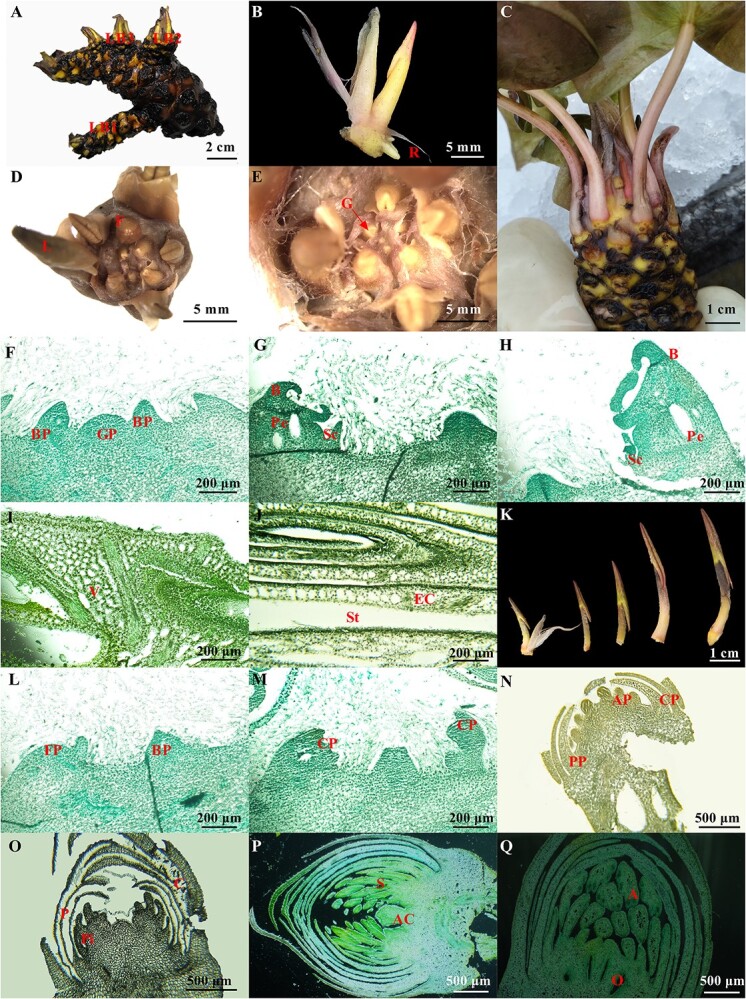
The morphology and anatomy of white water lily. (**A**) Morphological features of white water lily rhizome. LB1, LB2, LB3: lateral buds, labeled in order of initiation time. (**B**) Morphological features of the leaf bud. R: root tip. (**C**, **D**) Arrangement of leaf buds and flower buds. L: leaf bud; F: flower bud. (**E**) Growing point of the rhizome. G: growing point. (**F**) Leaf primordium stage. BP, bud primordium; GP, growth point. (**G**) Scale development stage. B, leaf; Pe, petiole; Sc, scale. (**H**) Young leaf formation stage. B, leaf; Pe, petiole; Sc, scale. (**I**, **J**) Mesophyll cell differentiation stage. V, veins; St, stomata; EC, epidermal cells. (**K**) Morphological features of the growing leaf buds; BP, bud primordia. (**L**) The early stage of floral bud differentiation. BP, bud primordia; FP, flower primordia. (**M**) The formation of the sepal. CP, corolla primordia. (**N**) The formation of petal and stamen primordium. CP, corolla primordia; AP, stamen primordia; PP, petal primordia. (**O**, **P**) The formation of the pistil and carpel primordium. P, petal; Pi, pistil primordia; C, calyx; AC: axial Prominence; S, sepal. (**Q**) The development and enlargement of floral buds. A, anther; O, ovary. Scale bars: A = 2 cm; B, D, E = 5 mm; C = 1 cm; F–J = 200 μm; K = 1 cm. L–M = 200 μm; N–Q = 500 μm.

The annual growth process of white water lily was first observed in this work. When temperature rises in the early spring (February–March), the floating leaves initiated rapidly from the dormant buds on the rhizome. The rhizome of the white water lily consists of underground stems from which the roots, leaves, and flowers emerge. It grows horizontally without obvious internodes (Fig. 1A). Adventitious roots form at the base of leaves. During this period, the submerged leaves retained throughout winter began to grow and gradually switched to the morphological characteristics of the floating leaves. Meanwhile, the floral buds formed before dormancy also began to sprout. As the apical bud grew away from the rhizome, adventitious roots developed at the ground-facing side of the bud. These roots exclusively formed at the base of the outermost leaves (Fig. 1B). With the progressive senescence of the outermost leaf, adventitious roots began to grow away from root primordia at the base of the inner leaves (Fig. 1B). Although root primordia formation occurred concomitantly with inner leaf initiation, its growth is suppressed until the outermost leaf began to senesce (Fig. 1B). The growing points were located within an area at the center of rhizome tips, where leaves and flowers originate (Fig. 1C–E).

The growth pattern of leaf buds of white water lily was the opposite and grew upwards in a spiral manner (Fig. 1C–E). The nascent leaf is folded and curled as a double cylinder with the leaf surface towards the center of the terminal bud, which is erect and pressed against the leaf petiole. There are scales on one side of the leaf, and there are hairs around the scales and leaf buds, which might play a protective role (Fig. 1B–D). As the leaves grow old and fall off, raised leaf scars, scales, and hairs remain on the rhizome. The hairs and scales eventually decay and detach over time, which might be the main cause of the blackened and fluffy appearance of the rhizomes (Fig. 1A). An axillary bud might exist in an old leaf axil, which would sprout and elongate in suitable conditions. The newly formed lateral stem also harbors growing points that can be detached for asexual reproduction (Fig. 1A).

The development process of white water lily leaves could be divided into the leaf primordium stage (Fig. 1F), the scale development stage (Fig. 1G), the young leaf formation stage (Fig. 1H), and the mesophyll cell differentiation stage (Fig. 1I and J). In the last stage, the divergence and formation of epidermal cells, stomata, and mesophyll tissue of the leaves are also completed (Fig. 1I). While the differentiation of mesophyll cells and vascular bundles is completed, the differentiation process of leaf bud basically ended, and an intact leaf bud was formed. After that, the leaf bud began to grow and uncoil and open fully before reaching the water’s surface (Fig. 1K).

From June to September, the white water lily entered the reproductive stage, and began flowering. The flowers fully opened in the morning and lasted for 3–4 days. After that, the flowers began shedding and gradually sinking into water as their pedicel became coiled. Flower bud differentiation occurred throughout the whole growth and development processes and could be divided into six stages: the early stage of differentiation (Fig. 1L), the formation of sepal (Fig. 1M), the formation of petal (Fig. 1N), the formation of stamen primordium (Fig. 1O), the formation of pistil and carpel primordium (Fig. 1P), and the development and enlargement of floral buds (Fig. 1Q).

**Figure 2:**
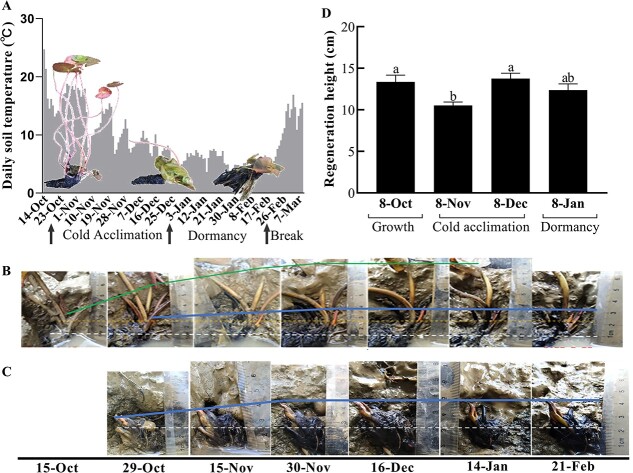
Growth dynamics of apical and lateral buds of white water lily. (**A**) Morphometric changes of whole white water lily. (**B**) Growth dynamics of apical buds. The upper and lower solid lines indicate the growth trends of old leaf buds and newly emerging leaf buds, respectively. The dashed line represents the central position of the rhizomes. (**C**) Growth dynamics of lateral buds. The solid line indicates the growth trends of newly emerging leaf buds, while the dashed line represents the central position of the rhizomes. (**D**) Regeneration height of leaves of rhizomes collected from different stages. Bars with different letters are significantly different (*P* < 0.05) according to Tukey’s multiple comparisons test.

White water lily began to form submerged leaves after October (Fig. 2A). Although there are still some slow-growing floating leaves, most of them became senescent, and the leaves on the water surface became scarcer (Fig. 2A). At this stage, white water lily initiated cold acclimation. After that, all the floating leaves of the white water lily disappeared until December, with a few persistent submerged leaves under water, and the growth of white water lily gradually stopped and entered the dormant stage (Fig. 2A).

**Figure 3:**
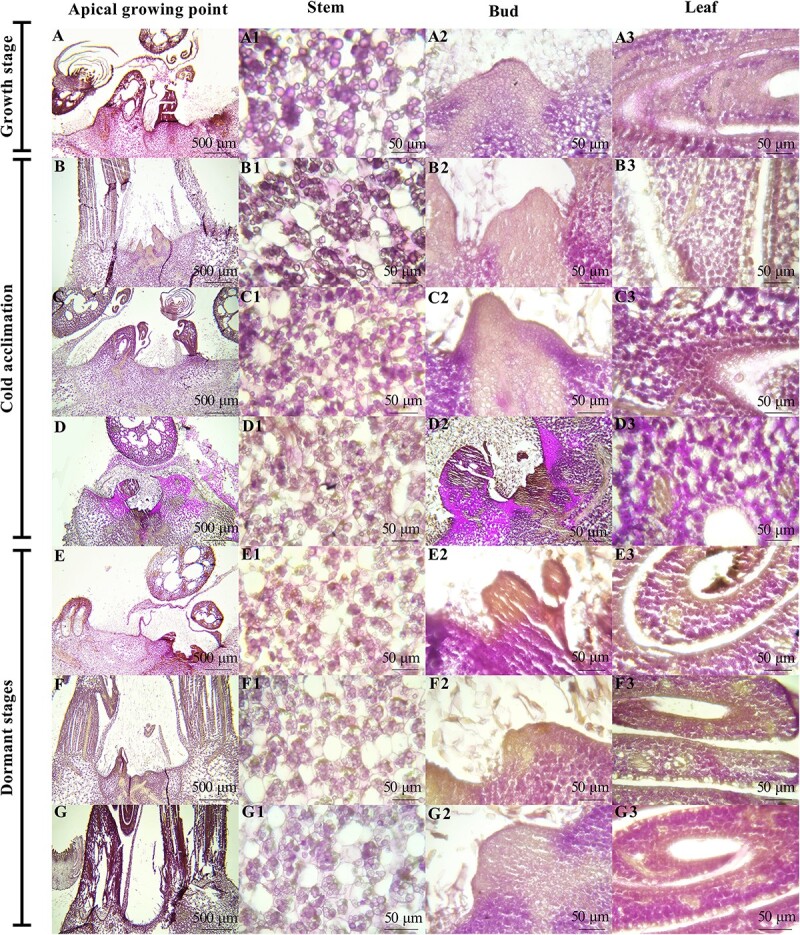
Histological analysis of paraffin sections of white water lily rhizomes during overwintering. (**A**) Anatomy of the rhizomes during growing stage, scale, 500 μm. (**B**–**E**) Anatomy of the rhizomes during cold acclimation stage. (**F**–**G**) Anatomy of the rhizomes during dormant stage. Apical growing point, scale: 500 μm; Stem, bud and leaf, scale: 50 μm.

As shown in Fig. 2B, the leaf buds in the growing points of the rhizome rapidly increased in size in the early cold acclimation stage while new leaf buds were also produced. At the late stage of cold acclimation, the newly emerging leaf buds began to grow as the old leaf bud growth slowed. When plants entered dormancy, all the leaf buds grew very slowly or even ceased growing (Fig. 2B). The leaf buds in the growing points of lateral rhizomes which had no newly emerging leaf bud also grew slowly in the stage of cold acclimation (Fig. 2C). It is found that the leaf buds in the lateral rhizome grew faster than the newly emerged leaf buds in the main rhizome, which indicated that the old leaf buds have a certain inhibitory effect on the growth of new leaf buds (Fig. 2C). Based on the rhizosphere temperatures, it was found that the white water lily entered the stage of cold acclimation at temperatures around 15°C, when the growth rate of water lily started to decline (Fig. 2). White water lily entered the dormant stage at the temperature down to around 10°C, when it slowed or even stopped growth (Fig. 2). In this process, the petiole of the old leaves gradually showed a water soaking phenotype and finally turned black and rotted, which could be caused by damaging cold temperatures (Fig. 2).

Rhizomes of water lily at the different stages were collected and put into water of 25°C after removing all floating leaves for growth resumption (Fig. 2D). The results showed that the rhizomes collected from the different stages exhibited rapid recovery in growth within one week, indicating that the dormancy status of white water lily was ecodormancy. The growth rate of rhizomes collected from cold acclimation and dormant stages was relatively slower than the rhizomes from the growth stage. It is interesting that the rhizomes from the early dormant stage could recover slightly more quickly than the rhizomes from the cold acclimation stage. Furthermore, we placed the dormant rhizomes of the white water lily and the tropical water lily ‘Golden State’ into a temperature of 4°C, which is representative of the conditions beneath ice layers in winter aquatic environments. We observed that the dormant rhizomes of ‘Golden State’ experienced a complete loss of germination ability within three months (Fig. S1, see online supplementary material). In contrast, under the same conditions, the dormant rhizomes of the white water lily exhibited a 100% survival rate for a duration exceeding four months, showcasing their superior cold tolerance.

### Histological analysis of paraffin sections of white water lily rhizomes

The results of histological analysis of paraffin sections showed that starch grains (stained a purple-red color) were widespread in the rhizomes, especially in the stem, leaves, and petioles (Fig. 3). The base of the buds could accumulate more starch granules at certain stages during overwintering. Protein (stained a pale-yellow color) was predominantly found in the growing points and the apices of buds (Fig. 3B). The amount of starch and protein continually underwent changes during dormancy and the change trends differed among the different parts of the rhizome. Based on the number and size of starch grains in cells, it was observed that starch content increased appreciably in the stem at the stage of incipient cold acclimation (Fig. 3A1 and B1), followed by a gradual, moderate decline at the later phase of cold acclimation until entering the dormant period (Fig. 3D1 and E1). In the early stage of cold acclimation, protein was not detected in the stem of white water lily until late in the stage (Fig. 3A1 and B1).

Changes in starch and protein contents in the shoot primordia and leaves exhibited the opposite trends (Fig. 3A2–G2). Protein was ubiquitously distributed in the vascular tissue of the leaf and in some stages was present in large amounts in the leaf upper epidermis (Fig. 3A3–G3). The fully developed flower buds were always observed in the rhizome during cold acclimation and dormant stages (Fig. 3).

The apical growth cones were mostly globose shape with obvious cell bumps during the dormant stage, and there were shoot primordia or the developing leaf buds around the apical growth cone, which indicated that the apical growth cone entered a quiescent state preserving high cellular viability (Fig. 3 F–G).

### Ultrastructural changes of white water lily during overwintering

**Figure 4:**
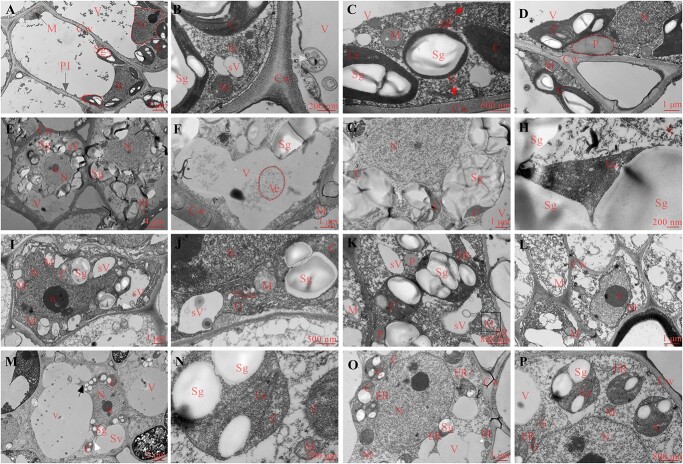
Ultrastructural changes in white water lily rhizome bud cells during overwintering. (**A**–**D**) Initial stage of cold acclimation. (A) The overall ultrastructure; (B) exocytosis or endocytosis events; white arrow indicates endocytosis events; (C) substructures of mitochondria and chloroplast; (D) the nuclei. (**E**–**H**) Late stage of cold acclimation. (E) The overall ultrastructure; (F) vacuolar inclusions; (G) starch grains in the chloroplasts; (H) chloroplast lamellar structure. (**I**–**L**) Dormant stage. (I) The overall ultrastructure; (J) the dissolved stromal lamellae; (K) the cristae appeared to be degraded; (L) incipient plasmolysis; the white arrow refers to the process that vesicles are engulfed into vacuoles; the black arrow refers to the location of incipient plasmolysis; the black box refers deformed mitochondria. (**M**–**P**) Recovery stage. (M) The overall ultrastructure; (N) chloroplast lamellar structure and mitochondrial cristae; (O) increased organelles surrounding the nucleus; (P) starch grains on chloroplasts; the white arrow refers the chloroplast division; the black arrow refers vacuolar fusion. Dashed lines are used to outline the organelles in the image at their first clear appearance. Scale bars: A, M = 2 μm, B, H, N = 200 nm, C = 600 nm, D, F, G, I, L, O = 1 μm, E = 4 μm, J = 500 nm, K, P = 800 nm. C: chloroplast; Cw: cell wall; ER: endoplasmic reticulum; G: golgi apparatus; La: lamellar structure; M: mitochondria; N: nucleus; P: plastid; Pl: plasmodesmata; Pm: plasma membrane; Sg: starch granules; SV: vacuole; V: large central vacuole; Ve: vesicles.

During the initial stage of cold acclimation of white water lily, the bud cells had a well-preserved cytoarchitecture (Fig. 4A). The plasmalemma was quite smooth, without apparent plasmolysis and with evidence of exocytosis or endocytosis events, which indicated that the cell metabolism of buds was relatively active at this time point (Fig. 4B). The central vacuole occupied most of the cell volume, with chloroplasts, mitochondria, endoplasmic reticulum, and Golgi around it (Fig. 4B and C). Clear and intact substructures of mitochondria and chloroplast such as cristae or lamellae could be observed. Some chloroplasts contained several starch grains in the chloroplast lamellae (Fig. 4C). The nuclei were located primarily at the periphery of the cell, with a well-defined nuclear membrane, evenly dispersed nucleoplasm and the clearly visible nucleolus (Fig. 4D).

In the late stage of cold acclimation, the ultrastructure of the buds showed that the nucleus moved towards the central region of the cells, while vacuoles moved towards the periphery of the cells and shrunk in size (Fig. 4E). At this time, mitochondria and chloroplasts were abundantly present in the cells, though cavities appeared in some mitochondria with a reduced number of mitochondrial cristae (Fig. 4F and G). Numerous starch grains accumulated in the areas around the nucleus and in the chloroplasts (Fig. 4G). The chloroplast structures gradually diminished and the chloroplastic membranes became blurred (Fig. 4H).

In the dormant stage, the nucleus occupied the majority of the cell central region and both the nucleus and nucleolus became larger (Fig. 4I). The large vacuole fragmented into smaller ones. The small vacuoles could still engulf the vesicles from the cytoplasm as evidenced by the presence of many clear vesicles in cells and the number of inclusions increased greatly (Fig. 4I). The number of chloroplasts was not only reduced but also stromal lamellae dissolved dramatically in the dormant stage (Fig. 4J and K). Meanwhile, the starch grains in the chloroplasts diminished in number and size. This indicated that the starch grains were continually hydrolyzed to soluble sugar, which could decrease the water potential of the cells to enhance cold resistance and provide energy to the cells. Although the number of mitochondria was not decreased, the cristae appeared to be degraded, being almost devoid of clear structures, or even empty (Fig. 4K). Small amounts of endoplasmic reticulum, Golgi, and plastids were also found in the cells. In addition, incipient plasmolysis was observed in some cells, indicating that the cells had incurred cold damage (Fig. 4L).

Subsequently, we placed the dormant rhizome of white water lily in a greenhouse to resume the growth. During the resumption growth, a series of changes occurred within the cells including the movement of the nucleus toward the edge of the cell (Fig. 4M), the regeneration of a single large vacuole by fusing small vacuoles, and the disappearance of plasmolysis. During this stage, chloroplast and mitochondria reformed, and we could observe the well-defined internal structure of them (Fig. 4N). The amount of Golgi and endoplasmic reticulum (ER) around the plasma membrane increased, which might provide the basis for the resuscitation of cold injured cells (Fig. 4O and P).

### Physiological changes of white water lily during overwintering

To evaluate physiological changes in the water lily during overwintering, the content of free proline, soluble proteins, soluble sugars, and MDA, and activities of antioxidant enzymes were analysed (Fig. S2, see online supplementary material). During the cold acclimation stage, the content of proline in different tissues increased continuously (Fig. S2A, see online supplementary material). Soluble protein contents in stems first decreased and then increased (Fig. S2B, see online supplementary material). The starch content in the stems continuously increased, while in the leaves, petioles, and buds, it initially increased but later decreased during cold acclimation. (Fig. S2C, see online supplementary material). The soluble sugar content in different tissues of white water lily at different stages demonstrated a tendency to increase first and then decrease (Fig. S2D, see online supplementary material). The role of osmoregulatory substances in ameliorating the impacts of cold stress is profound. Starch, for instance, serves a dual purpose: as an energy reserve during dormancy and, upon degradation, as a source of soluble sugars, especially vital when photosynthetic activity diminishes during advanced cold acclimation. The observed ebb and flow in soluble sugar content perhaps mirrors this metabolic shift.

The cold stress-induced accumulation of ROS and superoxide radicals pose a formidable challenge to cellular integrity. The resultant oxidative damage, especially to membrane systems, is a widely recognized hallmark of cold stress. During the intermediate stage of cold acclimation, the MDA content significantly increased in the stems, buds, and leaves of white water lily, which, however, decreased during the late stage (Fig. S2E, see online supplementary material). White water lily showed an overall increase in antioxidant enzyme activities (Fig. S2F–J, see online supplementary material). Polyphenol oxidase (PPO) activity in all tissues tended to increase at first and then decrease. The consistently low MDA levels in the rhizome buds suggest a protective mechanism, possibly facilitated by heightened antioxidant defenses or cellular compartmentalization, ensuring their survival during the overwintering phase. Further, the observed upregulation of antioxidant enzyme activities, especially during the peak winter month of December, signifies the plant’s proactive approach to counterbalance the ROS onslaught and maintain cellular homeostasis (Fig. S2, see online supplementary material).

### Cold acclimation response in white water lily entails a large transcriptome reprogramming

As no reference genome or transcript data have been reported for white water lily, we first generated a full-length transcriptome of white water lily via the SMRT full-length sequencing analysis. RNAs were isolated from various tissues of white water lily in different developmental stages, and then equally pooled for SMART-seq, yielding over 100 Gb of long reads and the gene discovery was nearly saturated within all size ranges. A total of 113 566 unigenes were obtained and 111 169 unigenes were annotated. Gene expression profiles of white water lily at initial, intermediate, and late stage of cold acclimation were analysed by high-throughput sequencing. A total of 422 985 600 raw reads were obtained from nine libraries, with an average of 46 998 400 raw sequencing reads and 46 711 971 clean reads per sample after the removal of low-quality reads (Table S1, see online supplementary material). Base sequence quality analysis showed that, in each sample, more than 92.68% of bases had quality scores higher than Q30, and 97.16% had scores higher than Q20 (Table S2, see online supplementary material). The 113 566 full length unigenes obtained from SMART-seq were used as reference transcripts to calculate the transcript abundance in each library.

The expression measurements from the three biological replicates for each library showed a strong correlation, with an average Pearson’s correlation coefficient value of 0.98 ± 0.01. This result demonstrates a strong correlation between the biological repeat data sets (Fig. S3A, see online supplementary material) and demonstrated a clear transcriptomic disparity between groups (Fig. S3B, see online supplementary material). The findings from the analysis of differential expression are presented in Table S3 (see online supplementary material), while the patterns of differential gene expression are visualized through scatter plots in Fig. S4 (see online supplementary material). At all three stages, a grand total of 5055 genes exhibited differential expression, highlighting the fundamental transcriptome reprogramming during overwintering.

To fully understand the function of these DEGs, Gene Ontology (GO) enrichment analysis was performed (Table S4, see online supplementary material). The top 20 most enriched GO terms of biological processes are exhibited in Fig. S5 (see online supplementary material). The most enriched GO terms in early cold acclimation were associated with genes involved in stress response, such as ‘cellular response to abiotic stimulus’ (GO:0071214), ‘defense response to fungus’ (GO:0050832), and ‘Antioxidant activity’ (GO:0016209). However, this differed starkly in the late stage of cold acclimation, where most differentially expressed genes were predominantly associated with gene transcriptional regulation, metabolite synthesis and other biological processes.

The Kyoto Encyclopedia of Genes and Genomes (KEGG) enrichment analysis revealed that the genes primarily regulated during the overwintering period are concentrated in metabolic pathways and biosynthesis of secondary metabolites (Fig. S6, see online supplementary material). The 6314 DEGs during the early stage of dormancy could map to 129 KEGG pathways and 37 enriched pathways were identified (Qvalue ≤ 0.05) (Table S5, see online supplementary material). Among the enriched KEGG terms throughout all stages of white water lily cold acclimation, the pathway of plant hormone signal transduction, amino acid metabolism, circadian rhythm emerged as a prominent feature.

The DEGs were categorized based on their shared expression patterns during cold acclimation, resulting in the identification of eight distinct expression profiles through expression trend analysis (Figs S7 and S8, see online supplementary material), providing further understanding of the complex molecular control underlying overwintering adaptation.

KEGG analysis revealed that pathways related to disease resistance, such as the plant-pathogen interaction and MAPK signaling pathway, gradually weakened as the temperature decreased, which was consistent with reduced activity of pathogenic microorganisms under low temperatures (Fig. S8, see online supplementary material). Genes involved in the photosynthesis pathway also gradually decreased with the reduction of leaf area.

As white water lily entered complete dormancy, the floating leaves disappeared, and the underground rhizomes lost a sufficient oxygen supply (Fig. S8, see online supplementary material). Consistent with this, many genes involved in aerobic respiration-related metabolic pathways, such as the citrate cycle (TCA cycle), and growth-related metabolic pathways, such as taurine and hypotaurine metabolism, as well as brassinosteroid biosynthesis were significantly decreased at the late stage.

The pathways related to porphyrin and chlorophyll metabolism, N-Glycan biosynthesis, and flavone and flavanol biosynthesis were activated only during the late stage of cold acclimation (Fig. S8, see online supplementary material). Throughout the cold acclimation stages, some pathways were continuously activated, such as plant hormone signal transduction, autophagy, ubiquitin-mediated proteolysis, proteasome, glycolysis/gluconeogenesis, ABC transporters, and basal transcription factors. It is interesting to observe that some growth-related metabolic pathways such as the pentose phosphate pathway and photosynthesis were activated at the end stage of cold acclimation, indicating that the dormant rhizomes seemed to begin preparation for rapid resuscitation.

Based on the results obtained, we have elucidated a comprehensive view of the dynamic transcriptional landscape of the white water lily under cold stress (Fig. S9, see online supplementary material). The observed upregulation of pathways related to plant hormone signaling, circadian rhythms, and metabolic processes indicates a broad recalibration undertaken by the plant to counteract the effects of decreasing temperatures. Notably, a significant increase in nitrogen metabolism, particularly in pathways related to amino acids, underscores the critical role of these compounds in enhancing cold resistance. Moreover, the accumulation of chaperone proteins, such as Heat Shock Protein 70 (HSP 70) and Late Embryogenesis Abundant (LEA), along with the activation of autophagy pathways observed in the white water lily, highlights sophisticated cellular strategies deployed to maintain protein homeostasis and integrity under cold stress conditions.

Contrastingly, the significant downregulation of genes associated with plant-pathogen interactions, mitogen-activated protein kinase (MAPK) signaling, and fatty acid metabolism during cold acclimation suggests a strategic shift in resource allocation under cold conditions. The reduced emphasis on certain metabolic and defense pathways, potentially attributable to diminished pathogenic activity at lower temperatures, indicates a reoriented biological focus during cold acclimation.

### Dynamic regulation of transcription factors during cold acclimation of white water lily

Our gene ontology (GO) term enrichment analysis underscored the pivotal role of transcription factor activity in the early adaptive response, enhancing the survival of the white water lily under cold conditions. Among the 301 DEGs encoding putative transcription factors, induced across various cold acclimation stages (Table S3, see online supplementary material), 44 are recognized as key regulators of plant cold tolerance (Fig. S10, see online supplementary material). Remarkably, the expression levels of 19 transcription factors consistently exceeded control levels throughout the cold acclimation process. These transcription factors primarily engage in the abscisic acid (ABA) signaling pathway (*ABA Responsive Element Binding Factor (ABF) 1*, *ABF4*, *ABA Insensitive (ABI) 5*), heat shock protein regulation (*Heat Shock Transcription Factor A (HSFA) 1*, *HSFA1A*, *HSFA1B*), circadian rhythm management (*Late Elongated Hypocotyl (LHY)*), and the integration of transcription regulation and signaling (*Alteration/Deficiency in Activation 2* (*ADA2*), *Arabidopsis Histidine Kinase 3* (*AHK3*), *APETALA2* (*AP2*), *BARELY ANY MERISTEM 8* (*BAM8*), *Basic Helix–Loop–Helix 62* (*BHLH62*), *Basic Leucine Zipper 1-A* (*BZIP1-A*), *CALMODULIN BINDING TRANSCRIPTION ACTIVATOR 5* (*CAMTA5*), *CYTOKININ RESPONSE FACTOR 1* (*CRF1*), *Ethylene Response Factor 2* (*ERF2*)). Additionally, six transcription factors showed phase-specific regulation, indicating their roles in the initial or terminal phases of cold acclimation. This dynamic adjustment aims to facilitate normal growth resumption or to prevent the growth inhibition caused by excessive response. These transcription factors, crucial to diverse signaling pathways and regulatory networks, are instrumental in the white water lily’s adaptation to cold environments. Moreover, we noted the downregulation of genes (*Basic Leucine Zipper 1* (*BZIP1*), *WRKY DNA-Binding Protein (WRKY) 33*, *WRKY76*, *BZIP60*, etc.) previously shown to boost cold tolerance, suggesting that the white water lily adopts unique strategies for cold acclimation. This downregulation might reflect a reduced reliance on these genes for cold tolerance or their diminished importance during specific adaptation phases.

### Phytohormones regulate cold acclimation response in white water lily

**Figure 5:**
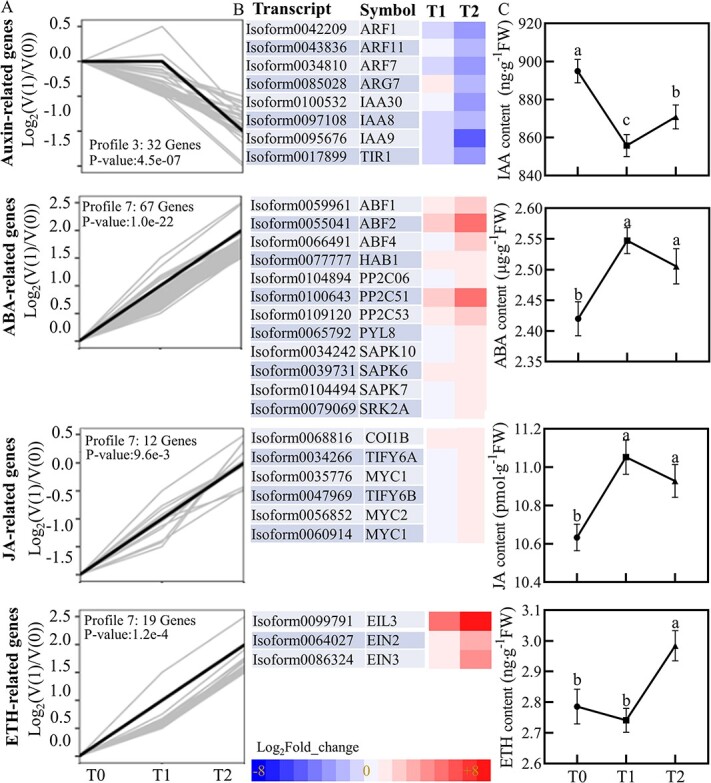
Dynamics of phytohormone levels and hormone-associated gene expression during cold acclimation in white water lily. (**A**) Expression profiles of phytohormone-related DEGs. The normalized expression profiles of phytohormone-related DEGs, categorized by their expression trends over the different cold acclimation stages T0 (initial stage), T1 (intermediate stage), and T2 (late stage). Each trend pattern is represented with individual genes displayed as gray lines, while the overarching trend for each group is highlighted with a black line. (**B**) Heatmap of DEGs encoding genes in the hormone regulation pathway across two time points, T1 and T2, relative to the control (T0). The color scale represents log2-transformed fold changes. (**C**) Phytohormone content in white water lily during cold acclimation. The value represents the mean ± SE of three independent biological repeats, and each experiment is repeated at least three times. Bars with different letters are significantly different (*P* < 0.05) according to Tukey’s multiple comparisons test. ABA, abscisic acid; ETH, ethylene; IAA, indole-3-acetic acid; JA, jasmonic acid.

In our study, the plant hormone signaling pathways were prominently featured among the enriched KEGG terms throughout the cold acclimation process of the white water lily, highlighting their crucial role in this adaptive mechanism. Therefore, we investigated which genes within the plant hormone signal transduction pathway (Ko04075) were responsible for these observations. An analysis of the expression trends of hormone-related genes (Fig. 5; Fig.  S11, see online supplementary material) was conducted alongside measurements of hormonal changes in the rhizome buds at different stages of cold adaptation (Fig. 5; Fig.  S12, see online supplementary material).

The analysis revealed a significant suppression of the auxin signaling pathway throughout the cold acclimation process, particularly impacting dormancy. Key regulatory factors in auxin signal response, such as *Auxin Response Factors (ARF) 1*, *ARF7*, and *ARF11*, which greatly influence cell division, elongation, and differentiation, were downregulated by 2.81-fold, 1.34-fold, and 2.53-fold, respectively, at the T2 stage compared to the T0 stage. Auxin-induced protein genes, including *Indole-3-Acetic Acid Induced Protein (IAA) 30*, *IAA8*, *IAA9*, and the auxin receptor gene *Transport Inhibitor Response 1 (TIR1)*, were downregulated by 2-fold to 4-fold during the dormancy stage (Table S3, see online supplementary material). The auxin content measurements indicated a rapid decline in the early stages of dormancy, followed by a slow increase upon entering the dormancy period.

Auxin, a critical hormone for promoting plant growth and development, undergoes a reduction in content and suppression of its signaling pathways as a mechanism for the plant to enter a state of dormancy, slowing down growth and metabolic activity. This response aids in reducing energy consumption while enhancing tolerance to low temperatures, likely representing an immediate reaction of the plant to cold stress to mitigate damage caused by environmental changes. The gradual increase in auxin levels after the plant has adapted to the initial cold environment and entered a state of dormancy could be aimed at maintaining essential life processes and facilitating rapid growth recovery when conditions improve. This observation aligns with the growth characteristics of the white water lily we observed (Fig. 2; Fig.  S1, see online supplementary material), suggesting a complex interplay between hormone regulation and environmental adaptation in plants.

In contrast, the signaling pathways of ABA, ethylene, and jasmonic acid (JA) appeared to be activated at different stages of cold acclimation. Within the ABA signaling pathway, the ABA receptor gene *PYR1-Like (PYL)8*, transcription factor encoding genes responsive to ABA, *ABA-Responsive Element Binding Factors (ABF) 1*, *ABF2*, *ABF4*, phosphatase genes responsive to ABA signals *Hypersensitive to ABA1 (HAB1)*, *Protein Phosphatase 2C (PP2C)06*, *PP2C51*, *PP2C53*, and kinase genes *Stress-Activated Protein Kinase (SAPK)10*, *SAPK6*, *SAPK7*, *Sucrose Non-Fermenting 1 (SNF1)-Related Protein Kinase 2A (SRK2A)* were continuously upregulated throughout the cold acclimation process (Fig. 2). Notably, *ABF2* and *PP2C51* experienced more than a five-fold increase at the T2 stage compared to the T0 stage. Measurements of ABA content showed a rapid initial rise in ABA levels during the early stages of cold acclimation, followed by a slow decline, maintaining at a certain level in the later stages.

The elevation of ABA levels during cold acclimation may prompt plants to reduce intracellular water loss, enhance membrane stability, and activate a series of genes associated with cold resistance. As the white water lily gradually adapts to the cold environment and potentially enters a state of dormancy, the stabilization and gradual decrease in ABA levels might reflect a slowdown in plant physiological activities (Fig. 2C). During dormancy, the plant’s growth and metabolic activities are reduced, likely diminishing the demand for ABA. Furthermore, the plant may have activated sufficient cold resistance pathways, eliminating the need for elevated ABA levels to maintain these responses. This dynamic change in ABA levels signifies its integral role in the adaptive response of white water lily during cold acclimation, aiding the plant’s survival in cold environments and preparation for winter dormancy.

JA and related signaling molecules play a pivotal role in the stress response of plants, particularly during the cold acclimation process [20], where they aid in plant adaptation to low temperatures by regulating gene expression, ultimately leading to dormancy. In our study, we identified key components within the JA signaling pathway and plant stress resistance responses, including members of the MYC gene family, *MYC1* and *MYC2*, as well as the key components of the JA signaling pathway, *Coronatine Insensitive 1B (COI1B)*, and TIFY family members *TIFY6A* and *TIFY6B*, which were continuously upregulated throughout the cold acclimation phase. Additionally, the expression of the JA signaling negative regulator, Jaz, was notably downregulated, potentially alleviating its suppression on JA signaling (Table S3, see online supplementary material). Analysis of hormone content in rhizome buds indicated that the trend of JA changes mirrors that of ABA, with both hormones initially rising rapidly and then slowly declining to maintain a certain level in the later stages. This suggests a synergistic role of ABA and JA in the white water lily response to cold stress, possibly by affecting the same signaling pathways or by regulating interacting target genes.

Core components of the ethylene signaling pathway, particularly *Ethylene Insensitive 2 (EIN2)* and *EIN3*, along with *Ethylene Insensitive 3-Like 3* (*EIL3*), were consistently upregulated during cold acclimation. A significant increase in ethylene content was detected in the later stages of cold acclimation, indicating the activation of the ethylene signaling pathway throughout this process. This activation likely interacts with other hormone signaling pathways, such as ABA and JA, forming a complex regulatory network to assist the plant in adapting to low temperatures.

The expression trends of genes within the cytokinin (CTK), gibberellin (GA), and salicylic acid (SA) signaling pathways were found to be complex, with some genes exhibiting stage-specific downregulation or upregulation. This indicates their multifaceted spatiotemporal roles during the overwintering process of the water lily. Additionally, a significant enrichment trend was observed for genes related to the brassinosteroid (BR) signaling pathway. Hormone content measurements in rhizome buds revealed that BR levels only increased during the later stages of dormancy. Similar to gene expression trends, the contents of GA, CTK, and zeatin riboside (ZR) also displayed varied trends (Fig. S12, see online supplementary material).

To further assess the roles of ABA and JA in enhancing the cold tolerance of waterlilies, we examined their effects on the cold tolerance of the tropical water lily ‘Golden Country,’ known for its particularly poor cold resistance. Following cultivation under low temperature conditions, leaves exhibited varying degrees of cold damage, as shown in Fig. S13 (see online supplementary material). The leaves of water lilies in the control group were the most severely affected, with entire leaves displaying water-soaked spots, the leaf tissue becoming transparent, and the veins becoming clearly visible. Evan’s blue staining revealed large, pale blue areas on the leaves (Fig. S13A, see online supplementary material), indicating widespread cell death. The electrical conductivity (EC) value for control plants was 36.21%, and the malondialdehyde (MDA) content was 0.018 μM/g FW (Fig. S13B and C, see online supplementary material). Plants treated with the hormones ABA and JA also showed varying degrees of water-soaked damage after cold treatment. However, they experienced milder cold injuries, smaller areas of water-soaked damage, and fewer Evan’s blue-stained areas (Fig. S13A, see online supplementary material). Compared to the control, pre-treatment with 20 μM ABA and 50 μM JA showed the best protective effects, with EC values decreasing by 8.52% and 7.08%, and MDA content decreasing by 33.33% and 27.78%, respectively (Fig. S13B and C, see online supplementary material). Data from molecular and physiological studies indicate that ABA and JA are involved in regulating the cold tolerance of the white water lily and offer similar protective effects in other waterlilies, suggesting their potential as agents to enhance the cold tolerance of tropical waterlilies.

Based on our results, we constructed a hormonal regulatory network diagram for the cold acclimation process in the white water lily. Our findings underscore the paramount roles of the ABA and JA pathways in the cold stress adaptation of waterlilies (Fig. S14, see online supplementary material), reflecting their well-documented roles across various plant species. The continuous activation of genes involved in ABA synthesis and signaling particularly highlights their crucial function during the cold adaptation process. The increase in ABA levels in the rhizomes, along with the regulation of SnRK2 and other ABA-responsive genes, further supports this point.

### Enhanced expression of circadian rhythm genes in white water lily cold response

The circadian clock deeply affects plant physiology and metabolism. Winter’s encroaching short daylight acts as a cue for numerous plants to commence their cold adaptation mechanisms. In our investigation involving the white water lily, we observed a significant upregulation in the majority of key genes within the classical circadian rhythm pathway during the initial stages of cold acclimatization (Fig. S15, see online supplementary material). Specifically, the core feedback loop components, such as *LHY*, exhibited augmented expression under cold stress. This upsurge subsequently amplified the expression of cold-responsive genes like *CBF* and *COR* [42], bolstering the plant’s adaptability to chilling conditions (Table S3, see online supplementary material). Another significant player in plant circadian clock is *GIGANTEA* gene (*GI*). Our observations during the water lily’s cold acclimation phase revealed a marked upregulation of GI (Table S3, Fig. S15, see online supplementary material). Even though GI does not influence CBFs’ expression, it augments the plant’s cold resilience by escalating soluble sugar content, thus ensuring optimal osmotic regulation [43]. This underscores GI’s intricate role in metabolic adjustments during low-temperature adaptation, profoundly influencing the plant’s resistance to cold.

We also identified *HY5* gene, a pivotal factor in light signal transduction and a critical component of the circadian clock signaling network, as being significantly upregulated (Table S3, Fig. S15, see online supplementary material). This upregulation appeared to be linked to an increase in various cold-responsive genes. Strikingly, we observed an enhancement in the expression of genes downstream of HY5 linked to anthocyanin biosynthesis, notably CHS, CHI, and FLS. Anthocyanins are renowned for their ability to shield plants from ROS-mediated damage during episodes of abiotic stress [44]. This suggests that they may play a role in augmenting the cold resistance capabilities of the water lily.

As dormancy progresses, certain genes within the circadian rhythm pathway undergo partial downregulation, demonstrating their association with the cold acclimation process (Table S3, Fig. S15, see online supplementary material). These results indicate that the circadian rhythm intricately engages in the white water lily’s cold adaptation. This involvement is a multifaceted interplay between light quality, the plant’s inherent stress responses, and, collectively, these factors work in harmony to bolster the water lily’s resilience to dropping temperatures.

### Metabolite profiling of white water lily during cold acclimation

The plant metabolome is often recognized as the bridge between the genome and phenome, as it fundamentally shapes the plant’s phenotype. Changes in the composition and content of metabolites are considered to be the final response of a plant to environmental stresses. Thus, we performed metabolome analyses of water lily during overwintering using a broadly targeted metabolomics approach. A total of 848 metabolites were identified.

**Figure 6:**
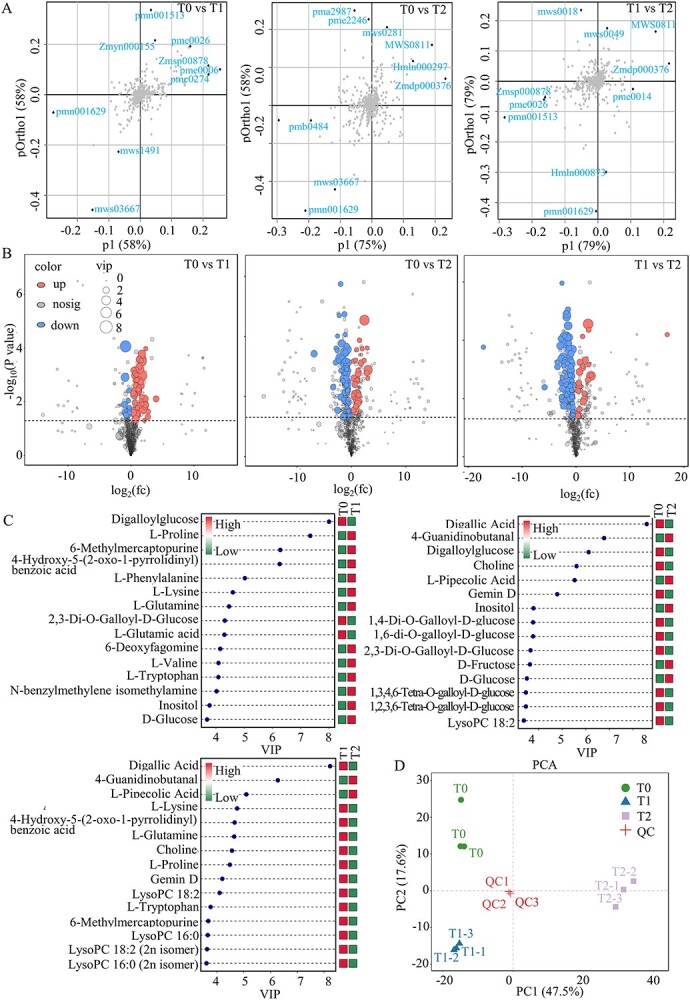
Metabolomics profiles of overwintering white water lily during cold acclimation stages. (**A**) OPLS-DA Score Plot. Variables distant from the origin on the X-axis contribute more significantly to distinguishing the sample groups. Variables in certain positions on the loading plot tend to have significant contributions to the samples located in the corresponding positions on the score plot, indicating that these are potentially key variables causing the differentiation. (**B**) Volcano plot of differential metabolites. The X-axis shows the log_2_-fold change in metabolite abundance across comparison groups, while the Y-axis represents the -log10 of the *P*-value from a *t*-test. The dashed line represents the *P*-value threshold for differential metabolite selection. Red dots represent metabolites with VIP ≥ 1 and *P* < 0.05 that are upregulated (FC > 1). Blue dots indicate metabolites with VIP ≥ 1 and *P* < 0.05 that are downregulated (FC < -1). The size of the dots indicates the VIP value of the metabolites. (**C**) The VIP plots of OPLS-DA. The X-axis represents the VIP values, while the Y-axis displays the top 25 differential metabolites. Metabolite abundance is averaged for each sample in the group, followed by a z-score analysis. The color on the right indicates the abundance level of this metabolite in different groups: red indicates upregulation, and green indicates downregulation. A larger VIP value suggests a greater contribution to sample differentiation, with metabolites having a VIP value larger than 1 being significantly different. CK, T1, and T2 denote samples collected from the rhizomes of white water lily during the initial, intermediate, and late stages of cold acclimation, respectively. (**D**) Principal component analysis (PCA) of metabolite profiles. The X-axis represents PC1, with the percentage in parentheses indicating the contribution of PC1 to the variation among samples. The Y-axis represents PC2, with the percentage in parentheses indicating its contribution to the variation. Colored dots represent individual samples; closer proximity of samples within a group indicates better repeatability.

Because metabolome data are characteristic of multidimensional nature and the variables are highly correlated, traditional univariate analyses are hard to efficiently mine the data. Therefore, we applied a chemometrics method and multivariate statistical techniques to perform a substantial dimensional reduction and classification of the multi-dimensional data. The X matrix information was decomposed into components connected and unconnected with the Y matrix (Fig. S16, see online supplementary material). Relevant information was then presented in the first predictive component after removal of variation not correlated to the metabolite changes (Fig. 6A). Based on a combination with the VIP indices provided by OPLS-DA (VIP ≥ 1) and a univariate *P*-value cutoff from a *t*-test (*P* < 0.05), we screened differential metabolites for each group and presented them in Fig. 6B. In contrast to the initial stage of cold acclimation, there were 68 metabolites that exhibited differential accumulation during the early stage, of which the content of 55 metabolites increased and 13 decreased (Fig. 6B; Table S6, see online supplementary material). The difference increased to 114 metabolites during the late stage, of which the content of 24 metabolites increased and 90 decreased. Several differential metabolites have been shown to participate in plant cold acclimation.

Based on the VIP value obtained from OPLS-DA, we measured the importance of the metabolites and the contribution of them in the sample discrimination. Fig. 6C showed the 15 dominant components in each comparable groups according to their VIP scores. Those metabolites were clustered per class into polysaccharides, sugars, sugar alcohol, phenolic acids, purine, alkaloids, amino acids, and some of its derivatives. Four of them were upregulated metabolites including D-glucose, inositol, L-proline, and betaine. Eleven metabolites were downregulated, among which the most diverse were some amino acids and their derivatives. Principal component analysis (PCA) of the metabolome data showed tight clustering of replicate samples, confirming the reproducibility of the results (Fig. 6D).

**Figure 7:**
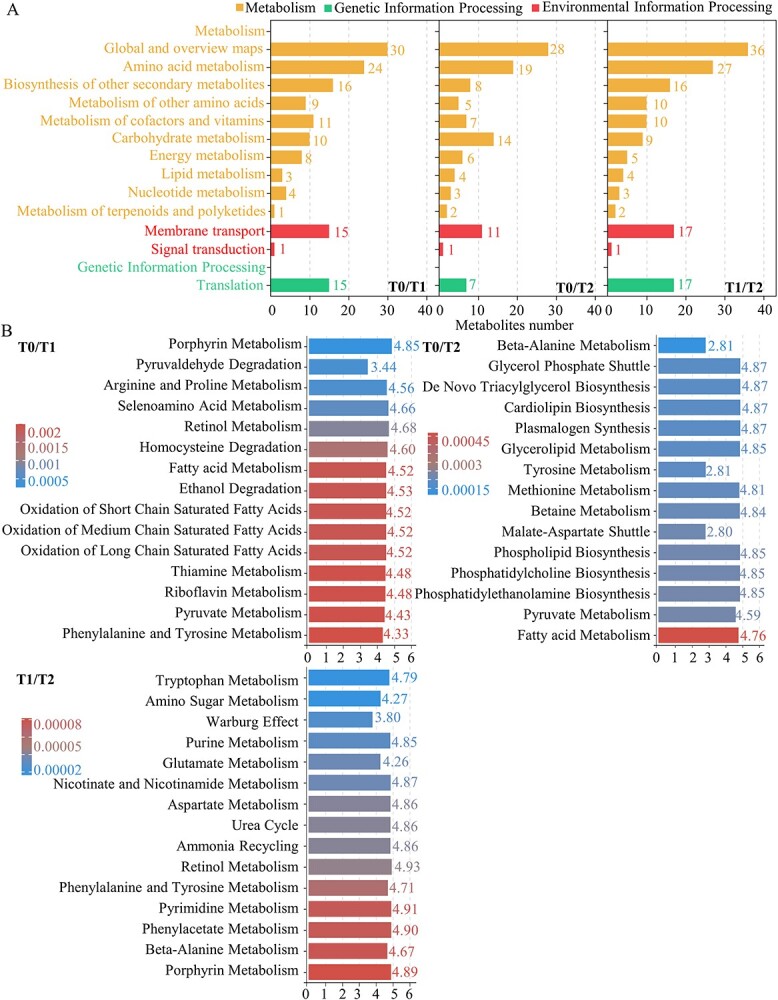
KEGG enrichment and MSEA analysis of differential metabolites. (**A**) KEGG enrichment analysis of differential metabolites. The graph represents different pathways and their respective numbers of target metabolites. Pathways belonging to the same KEGG A and KEGG B classifications are colored similarly. Each bar represents a pathway, and its length signifies the number of metabolites it contains. The legend denotes the specific pathways each color represents. (**B**) MSEA (Metabolite Set Enrichment Analysis) of differential metabolites. The Y-axis indicates the names of enriched metabolite sets, while the X-axis represents the degree of enrichment, calculated as the Enrichment ratio = Statistic Q/Expected Q. Bar colors signify the *P*-value magnitude, with the top 25 significantly enriched metabolite sets arranged in ascending order based on *P*-values. Darker shades indicate smaller *P*-values.

KEGG enrichment analysis revealed that many metabolic pathways, such as amino acid transport and metabolism, secondary metabolite biosynthesis, and carbohydrate transport and metabolism, were overrepresented (Fig. 7A).

To further explore the pattern of metabolites changes in some important pathways, we performed a global test analysis using the quantitative enrichment analysis (QEA) mode of Metabolite Set Enrichment Analysis (MSEA) for metabolites of all samples in each comparison group (Fig. 7B). MSEA indicated that fatty acid metabolism and respiratory metabolism, including ‘Fatty Acid Metabolism’, ‘Ethanol Degradation’, ‘Mitochondrial Beta-Oxidation of Short Chain Saturated Fatty Acids’, ‘Mitochondrial Beta-Oxidation of Medium Chain Saturated Fatty Acids’, ‘Mitochondrial Beta-Oxidation of Long Chain Saturated Fatty Acids’, ‘Riboflavin Metabolism’, and ‘Pyruvate Metabolism’ were highlighted throughout the cold dormant stage.

We then investigated the impact of three significant differential metabolites—betaine, myo-inositol, and proline—on the cold tolerance of water lilies (Fig. S13, see online supplementary material). Our results revealed that treatment with different concentrations of proline (Pro) demonstrated that 200 μM Pro offered the most effective protection. Following cold stress, leaves treated with this concentration exhibited less severe water-soaked damage and smaller areas stained with Evan’s blue, indicating reduced cellular damage and enhanced tolerance to cold stress.

Similarly, water lilies pre-treated with varying concentrations of betaine showed noticeable water-soaked damage after cold stress (Fig. S13A, see online supplementary material); however, the damage was less severe compared to the control. Treatment with 1 mM betaine significantly reduced the EC and MDA content in water lily leaves (Fig. S13B and C, see online supplementary material), suggesting that exogenous betaine application could mitigate cold stress-induced damage. This aligns with betaine’s role as an osmoprotectant, enhancing plant resilience to abiotic stress by stabilizing cellular structures and functions.

Most notably, leaves pre-treated with myo-inositol and subjected to cold stress exhibited no apparent water-soaked damage nor were they stained by Evan’s blue (Fig. S13A, see online supplementary material). Among all treatment groups, the 3 μM myo-inositol pre-treatment group showed the lowest EC and MDA values, at 22.49% and 0.01 μM/g FW, respectively (Fig. S13C and D, see online supplementary material). These values were significantly lower than those of the control group, showing reductions of 13.72% and 42.35%, respectively. This indicates that myo-inositol pre-treatment significantly enhanced cold tolerance in water lilies, playing a crucial role in their adaptation to colder conditions. Myo-inositol’s protective mechanism is likely through its function in membrane stabilization and as a signaling molecule modulating stress-responsive pathways.

### Cold acclimation in white water lily plants results in accumulation of amino acids and mobilization of antioxidant mechanisms

As transcriptional and metabolic responses were evidently not independent of each other, we further performed association analysis of transcriptome and metabolome based on three types of models including the pathway functional model, O2PLS (bidirectional orthogonal projections to latent structures), and the correlation coefficient model. Analysis of shared KEGG pathways between differential genes and metabolites showed that 66 and 58 KEGG pathways were significantly enriched during the early cold acclimation stage, and the late cold acclimation stage respectively. Additionally, 55 KEGG pathways were co-enriched in both stages, including five pathways related to amino acid metabolism that were also enriched among the differential genes (Table S7, see online supplementary material). O2PLS was used to integrate plant transcript and metabolite data. The number of components within each group was estimated by the cross-validation method, and the model with the lowest prediction error was chosen for subsequent analysis (Tables S8 and S9, see online supplementary material).

**Figure 8:**
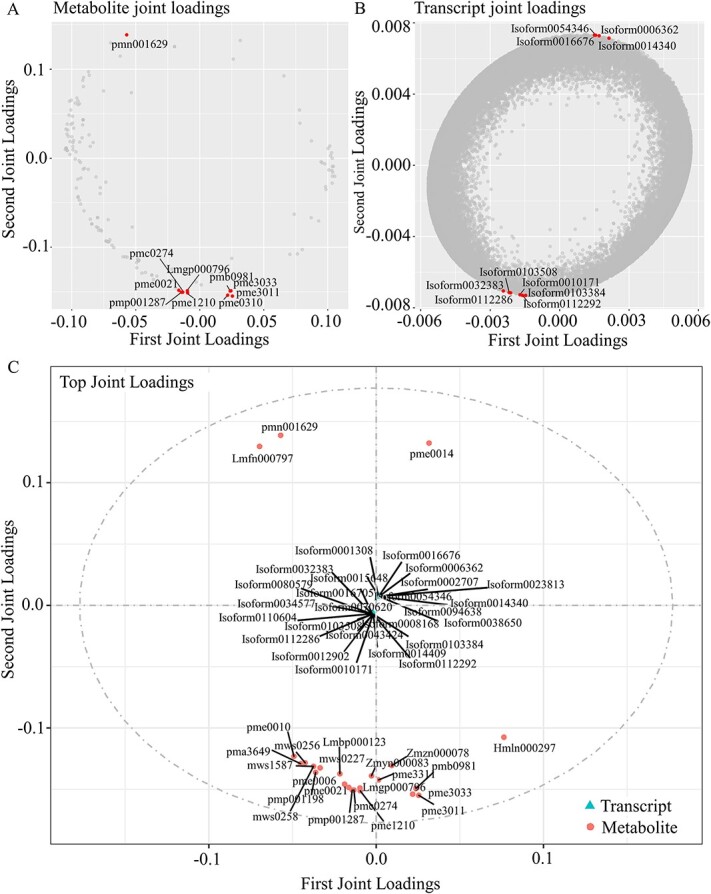
Metabolite and transcript joint Loadings plots. (**A**) The O2PLS loadings plot of metabolites. (**B**) The O2PLS loadings plot of transcripts. (**C**) The association loadings plots. The sign of the loading value indicates positive or negative correlation with another omics data type. The absolute value of the loading value indicates the strength of the correlation.

We generated separate joint loadings plots for different omics data types (Fig. 8A), which showed the explanatory power of variables (metabolites/genes) in each component, indicating their contribution to the differences between groups. The larger the absolute value of the loading value, the stronger the correlation. Based on the results of the loading values (Tables S10 and S11, see online supplementary material), the top 25 genes and metabolites with the highest sum of squared loading values for the first two dimensions were selected to generate an integrated loadings plot to display the genes and metabolites with the highest degree of correlation. The results showed that ten of the top 25 metabolites with the largest association were amino acids and their derivatives (Fig. 8B). This suggested that the amino acid metabolism was a crucial regulatory mechanism for waterlilies during the overwintering phase. Based on the changes in the content of metabolites and the expression level of genes related to amino acid metabolism, we mapped the central metabolic pathways of related amino acids (Fig. S17, see online supplementary material). During the cold acclimation process, the levels of glutamine, phenylalanine, and tryptophan were significantly reduced, while the level of proline was significantly increased.

By calculating the Pearson correlation coefficient between gene expression levels and metabolite abundance, we evaluated the correlation between genes and metabolites. We filtered out the top 250 differentially expressed genes and/or differential metabolites with an absolute correlation coefficient greater than 0.5 and constructed a network diagram (Fig. S18, see online supplementary material). The network diagram of the correlations revealed several genes and metabolites that occupy important positions in the correlations, such as zmdp000376, pmb0964, mws0191, and zmhn003397.

Phosphoenolpyruvate (PEP) is a critical precursor in the biosynthesis of aromatic amino acids (Fig. S17, see online supplementary material). During the cold acclimation process in the white water lily, a suite of DEGs including *phosphoglucomutase 1* (*PGM1*), *phosphoglycerate mutase* (*PGAM*), *6-phosphofructokinase* (*PFK*), *transaldolase* (*Tal*), *3-deoxy-D-arabino-heptulosonate 7-phosphate synthase* (*DAHPS*), *arogenate dehydrogenase* (*AroDE*), and *shikimate kinase 2* (*SK2*) regulated the conversion of PEP into branch acids. This conversion marks the bifurcation point for the synthesis of tryptophan as well as tyrosine and phenylalanine. The synthesis of tryptophan was negatively regulated by the Trp operon, particularly the expression of *TrpE*, through a negative feedback mechanism reducing the production of tryptophan and thereby limiting the conversion of branch acids to tryptophan. In the biosynthesis pathways of phenylalanine and tyrosine, the branch acid isomerase (CM) facilitated the conversion of branch acids into prephenate, which subsequently forms tyrosine or phenylalanine.

Phenylalanine, tyrosine, and tryptophan, as core components of plant metabolism, play essential roles in plants’ defense against various biotic and abiotic stresses. Phenylalanine, in particular, serves as a precursor for many multifunctional secondary metabolites. Its derived volatile compounds are involved in plant reproduction and defense responses, contributing to enhanced stress resistance. The decrease in phenylalanine content during cold acclimation was associated with its role in plant stress resistance [21]. Exogenous application of phenylalanine has been reported to enhance cold tolerance in tomatoes by activating the ROS scavenging system. Additionally, cold stress leads to an increase in phenylalanine content, further underscoring its significant role in plant adaptation to environmental stress.

Pyruvate, a crucial end product of the glycolytic pathway, not only participates in the synthesis and metabolism of amino acids but also serves as a common intermediate in the metabolism of six major amino acids: alanine, cysteine, serine, glycine, threonine, and tryptophan (Fig. S17, see online supplementary material). The conversion of PEP to these amino acids, further promoting the synthesis of pyruvate, could be induced by the expression of *pyruvate kinase* (*PK*). However, under cold stress conditions, the metabolic capacities of amino acids such as tryptophan, glycine, and threonine were reduced due to the inhibitory actions of genes like *serine hydroxymethyltransferase* (*SHMT*) and *threonine aldolase 1* (*THA1*), consequently suppressing pyruvate synthesis. Moreover, the catabolism of tryptophan generated a variety of indole-containing secondary metabolites. Auxin, a key metabolite synthesized from tryptophan, significantly influences plant growth and stress response.

Glutamine plays multiple roles within plant systems, including participation in the formation of aminosugars, glutathione, L-glutamate, and other amino acids, protein synthesis, and glucose production [22]. During cold acclimation of white water lily, glutamine levels were significantly upregulated, whereas they were downregulated in the later stage. This fluctuation was regulated by glutamate synthase (GLT1) and glutamine synthetase (GlnA), enzymes responsible for the interconversion between glutamine and glutamate. Transcriptomic data revealed that the expression of the glutamine synthetase gene *GlnA*, related to proline synthesis, is downregulated during dormancy, suggesting a reduction in glutamine synthesis and a relative increase in glutamate content during this period.

In cold environments, plants predominantly produce proline through the glutamate pathway, an amino acid converted from ornithine and glutamate, regulated by genes such as *N-acetylglutamate kinase* (*NAGK*) and *N-acetylglutamate synthase* (*NAGC*). Furthermore, glutathione, which can be synthesized from glutamine under the regulation of *GST* genes, experienced inhibited conversion due to downregulation of *GST* expression. Glutathione is closely associated with the plant’s antioxidative capacity, thus this metabolic change aids in enhancing plant antioxidant properties [23].

Our results indicate that the white water lily enhances its resistance to cold environments by regulating amino acid metabolism. Particularly, proline becomes a focal point of research due to its significant accumulation under adverse conditions such as cold stress, producible via two biosynthetic pathways: one derived from glutamate and the other from ornithine. In this study, the upregulation of proline levels and its exogenous application were shown to enhance the cold tolerance of water lilies (Fig. S13, see online supplementary material), suggesting that its accumulation contributes to increased plant cold resistance. Similarly, chrysanthemums also exhibited proline accumulation following cold acclimation, thereby enhancing their tolerance to cold damage.

## Discussion

The alpine terrains of Xinjiang, China, harbor a myriad of botanical wonders, with the white water lily being one of the most remarkable. Its inherent ability to flourish amidst the region’s stringent cold challenges underscores its resilience and places it in a unique pedestal within the water lily genus. Its potential as a touchstone for cold tolerance breeding in the water lily community is undeniable. Nevertheless, despite its exceptional stature, the intricate processes and the myriad mechanisms driving its cold resistance have remained enigmatic until recently.

In our pursuit to decipher the multifaceted adaptations employed by the white water lily during overwintering, we amalgamated diverse analytical paradigms. Ranging from morphological assessments to probing molecular intricacies, our investigative lens sought to cover the full spectrum of the plant’s response to cold acclimation. Our findings reveal that this species could maintain high cellular vitality and growth potential during winter, demonstrating a robust resistance to cold temperatures. This resilience is achieved through an effective reallocation of resources and morphological adjustments, including compartmentalization in large vacuoles, accumulation of osmoregulatory substances, and enhanced antioxidant capacities. The pivotal role of plant hormone signaling, amino acid metabolism, and circadian rhythms in mediating cold adaptation strategies underscores the complexity and sophistication of plant responses to abiotic stress.

The white water lily displays unique morphological characteristics, such as the asymmetric development of side buds and distinct root formation at the leaf base, which are uncommon among other tuberous plants. Central to their winter survival is the vitality and robustness of their rhizomes. The observed horizontal growth pattern of the underground rhizomes and the development of side buds into side shoots draw parallels to some established botanical phenomena seen in gingers [24]. However, the water lily exhibits a divergence in its side shoot development, following an asymmetric pattern, in contrast to the symmetrical growth found in ginger. As temperatures decline, the gradual deceleration of leaf growth and subsequent dormancy underpin an adaptive resource reallocation strategy. By conserving resources and diverting them towards enhancing cold resistance, the white water lily epitomizes an evolutionary trade-off between growth and survival.

Our ultrastructural investigations into the rhizome cells of waterlilies during overwintering unveiled a dynamic interplay of subcellular modifications. Key among these is the restructuring of vacuoles, which along with increased intracellular content, appears to be a crucial adaptation to prevent cold injury.

The strategy to curtail cellular water content, reminiscent of the responses observed in cotton leaves under cold stress [25], further affirms the universal adoption of such defensive mechanisms across plant species. Additionally, the alterations in chloroplasts and mitochondria highlight the sensitivity of these organelles to cold stress, which aligns with prior findings in other plants [26].

The intricate dynamics of cold acclimation in plants, as epitomized by the water lily rhizome, highlight a multifaceted interplay of morphological, physiological, and biochemical shifts. As daylight duration decreases and temperatures decline, plant hormone signaling pathways, genes related to circadian rhythm regulation and some cold-related transcription factors rapidly initiate signaling at the onset of cold acclimation. Central to this is the modulation of osmoregulatory substances and antioxidant defenses, which collectively orchestrate a robust response to cold stress. The role of osmoregulatory substances in ameliorating the impacts of cold stress is profound. These compounds functionally decrease the cell water potential, consequently lowering the freezing point of cellular fluids and bolstering the plant’s resistance to cold stress. The prominence of starch, soluble sugars, soluble proteins, and free proline as pivotal osmolytes in this context is well documented [27]. Starch, for instance, appears to serve a dual role: firstly, as an energy reservoir to sustain the plant during dormancy, and secondly, upon its degradation, as a source of soluble sugars. The latter is particularly crucial as the onset of reduced photosynthetic activity in the advanced stages of cold acclimation necessitates the mobilization of stored energy to fuel life-sustaining processes. The observed ebb and flow in soluble sugar content perhaps mirrors this metabolic shift, aligning with findings from prior research [45]. Nitrogen-containing compounds, crucial for plant growth and development, seem to play an intensified role under cold stress. This aligns with observations in other species wherein amino acid metabolism is a pivot for cold resistance, supplying both osmoprotectants and precursors for protein synthesis [28]. The augmentation in proteins and free proline content during the cold acclimation period in the white water lily hints at their indispensable roles in cellular osmoregulation and cold adaptation. Beyond their conventional cellular functions, these compounds are instrumental in mitigating the osmotic imbalances induced by cold stress. The empirical evidence underscoring the role of proline, in particular, is compelling. The marked enhancement of cold resistance in tropical water lilies upon exogenous proline addition affirms its centrality in cold acclimation strategies.

Metabolite profiling has shed light on specific compounds contributing to cold tolerance. Notably, myo-inositol, known for its pivotal role in various stress responses and cellular processes [29], was prominently upregulated during cold acclimation. Our experimental evidence pointing towards the protective role of exogenously applied myo-inositol further emphasizes its potential as a cryoprotectant in waterlilies. Similarly, the observed increase in betaine, a renowned osmoprotectant, aligns with previous findings emphasizing its role in stress responses [46]. The clear enhancement in cold resistance upon its external application to tropical waterlilies underlines its potential application in enhancing plant cold resistance.

Concurrently, the cold stress-induced accumulation of ROS and superoxide radicals pose a formidable challenge to cellular integrity. The resultant oxidative damage, especially to membrane systems, is a widely recognized hallmark of cold stress [47]. The intriguing MDA content dynamics across different parts of the white water lily during overwintering offer valuable insights into its oxidative stress responses. The consistently low MDA levels in rhizome buds suggest a protective mechanism, possibly facilitated by heightened antioxidant defenses or cellular compartmentalization, ensuring their survival during the overwintering phase. Further, the observed upregulation of antioxidant enzyme activities, especially during the peak winter month of December, signifies the plant’s proactive approach to counterbalance the ROS onslaught and maintain cellular homeostasis.

In our current endeavor, we embarked on a multifaceted examination of the white water lily’s response mechanisms during overwintering. Our analyses not only charted the physiological evolution of the plant under cold stress but also mapped the perturbations at the transcriptomic and metabolomic levels. The integration of these data types provided us a panoramic view of the interlocking mechanisms the water lily leverages to fortify its cold resistance, particularly during its critical overwintering phase. To encapsulate this complex web of interactions, we distilled our insights into a coherent model, as illustrated in Fig. S19 (see online supplementary material).

During overwintering, the white water lily demonstrates effective resource reallocation to bolster its resistance against cold, facilitated by morphological adjustments. Key to this adaptation is the strategic compartmentalization of large vacuoles, alongside the accumulation of osmoregulatory substances, and an increase in antioxidant capacity. A complex molecular regulatory network forms the foundation of the white water lily’s hardiness during winter. This network prominently features phytohormone signaling, amino acid metabolism, and circadian rhythms as key elements in the plant’s defense against cold. Our findings highlight the vital role of nitrogen metabolism, particularly amino acid-related pathways, and the significance of compounds such as myo-inositol and L-proline in enhancing cold tolerance. These components collectively form the core of the plant’s adaptive strategy, effectively coordinating the morphological, developmental, and physiological responses that are critical for its survival in the harsh alpine environment.

Additionally, our research uncovered unique idiosyncrasies in the white water lily. While its cold acclimation strategies predominantly conform to established pathways observed in other plant species, we identified significant deviations. A notable finding is the subdued role of unsaturated fatty acids in the water lily’s temperature regulation, contrasting with their recognized importance in other plants for modulating membrane fluidity and facilitating cold resistance. This minimal involvement of unsaturated fatty acids in the water lily’s adaptation strategy suggests the presence of alternative, yet-to-be-explored compensatory mechanisms. These insights not only deepen our understanding of plant adaptive mechanisms but also lay the groundwork for further research. The peculiarities observed in the water lily, set against the context of conserved adaptive responses, raise compelling questions: What alternative mechanisms does the white water lily employ to obviate the need for unsaturated fatty acids? Could these alternative strategies offer more effective or resource-efficient solutions, potentially providing valuable insights for plant biology and agricultural practices?

Delving deeper into the cold acclimation intricacies, it becomes palpably clear that this is not a singular, linear response. Instead, it is an orchestrated symphony of molecular cues and cellular pathways. Our data indicate that these elements are not just passive components but active participants, intricately shaping the robust cold resistance of the white water lily during overwintering. These findings offer novel perspectives for our understanding of plant adaptive mechanisms under low-temperature stress.

## Materials and methods

### Plant materials and growth conditions

For this study, two species of water lilies were utilized: White water lily (*N. candida*) and tropical water lily (*N. thermarum* ‘Huangjingguo’). The seedlings of these water lilies were initially planted in planting bags with a diameter of 380 mm. These bags were subsequently placed inside larger pots, each having a diameter of 535 mm, during the month of April. The watering regime was carefully managed to align with the distinct growth stages of the water lilies. Initially, a moderate amount of water was introduced to the pots. As the plants grew, the water level was incrementally increased, eventually resulting in the pots being completely filled with water.

The experimental design incorporated three to five biological replicates for each treatment condition, wherein each replicate consisted of a composite sample derived from a minimum of three individual water lily plants. Sample collection was consistently performed in the mornings between 9:30 and 11:00 to minimize the potential variations caused by diurnal changes.

### Chemicals and reagents

In this research, every chemical and reagent employed was ensured to be of analytical quality. We sourced ethyl alcohol, methyl alcohol, and acetonitrile specifically from the Merck Company in Germany. For the purpose of obtaining ultrapure water, we utilized the Milli-Q system provided by Millipore Corp., Bedford, MA, USA. Moreover, standard substances were obtained from two sources: BioBioPha Co., Ltd and Sigma-Aldrich, located in the USA.

### Preparation of paraffin sections

After cleaning, the plant samples were fixed in a 70% FAA solution and processed through several steps to produce paraffin sections, including dehydration, clearing, wax infiltration, embedding, sectioning, mounting, and coverslipping [30]. The paraffin sections were stained using the safranin-fast green staining method [31]. Furthermore, an improved periodic acid-Schiff (PAS) reaction method was employed to stain starch and proteins in the samples. This staining method allowed the observation of morphological changes in the rhizomes during overwintering, as well as the changes in starch and protein content [32].

### Transmission electron microscopy observation

The samples were quickly rinsed, cut into suitable sizes, and placed in a 2.5% glutaraldehyde solution for fixation. Once the samples were adequately fixed, they underwent processing and sectioning following the methods described for TEM sample preparation and processing [33]. Subsequently, the prepared sections were observed for cellular ultrastructure using a Hitachi H-7650 transmission electron microscope.

### Analysis of physiological indices

Sampling was conducted during the cold acclimation stages of white water lily. The collection was carried out between 9:30 and 11:00 in the morning. At least three randomly selected whole plants were excavated for each treatment. After cleaning the plants, any decayed or residual tissues were removed. The whole plants were then dissected into several parts, including leaves, petioles, buds, and stems, and used for the analysis of soluble sugars, soluble proteins, starch, free proline, MDA, antioxidant enzymes, and phytohormone content. Soluble sugar content was determined as in Zhang *et al.* [34]. A Solarbio Detection Kit was used to determine starch content [35]. The MDA content was quantified utilizing the procedure described by Jin *et al.* [36]. We extracted and measured the free proline content in the tissues following the protocol detailed in Li *et al.* [37]. For the extraction and analysis of total soluble protein content, we adhered to the methodology proposed by Barretto *et al.* [38]. The phytohormone content in rhizomes of white water lily was detected using the ELISA kit from YAFEIBIO.

### Transcriptome analysis

During the initial, intermediate, and late stage of cold acclimation, rhizomes of white water lily were sampled for transcriptome analysis. To minimize diurnal variations’ impact on gene expression, sample collection was strictly performed between 9:30 and 11:00 a.m. For each stage, three independent biological replicates were obtained. Each replicate comprised a composite of at least three individual samples to reduce biological variability in transcriptome studies. Upon collection, samples were immediately flash-frozen in liquid nitrogen to prevent RNA degradation. The frozen samples were then stored at −80°C until RNA extraction. Total RNA extraction and quality assessment were conducted using TRIzol reagent and Agilent 2100 Bioanalyzer, respectively (Agilent Technologies, Palo Alto, CA, USA) and verified with RNase-free agarose gel electrophoresis. Eukaryotic mRNA was enriched selectively using Oligo(dT) beads with the Ribo-ZeroTM Magnetic Kit (Epicentre, Madison, WI, USA). The mRNA samples were then fragmented into smaller segments using fragmentation buffer and reverse transcribed into cDNA using random primers. This was followed by the synthesis of second-strand cDNA. The cDNA fragments underwent purification with the QiaQuick PCR extraction kit (Qiagen, Venlo, The Netherlands), followed by end-repairing, addition of poly(A) tails, and ligation with Illumina sequencing adapters.

Post-ligation, size selection was conducted via agarose gel electrophoresis, and the products were amplified through PCR. The final stage involved sequencing the cDNA libraries using Illumina HiSeqTM 4000, conducted by Gene Denovo Biotechnology Co. (Guangzhou, China). Raw reads were initially processed using fastp for quality filtering. The clean reads were then aligned to the reference transcriptome via RSEM [39]. Differential expression analysis of RNAs between groups was executed using DESeq2 [40], considering genes with a false discovery rate (FDR) less than 0.05 and an absolute fold change of 2 or more as differentially expressed. Protein–Protein interaction network was deduced using String v10 [41].

### Metabolomic analysis

The rhizomes of white water lily were first lyophilized and then pulverized using a Retsch MM 400 mixer mill with zirconia beads for 90 seconds at 30 Hz. We then extracted 100 mg of this powdered sample in 1.0 ml of 70% methanol, supplemented with 0.1 mg/L lidocaine as an internal marker, at 4°C overnight. Post-extraction, the mixture was centrifuged at 10000 *g* for 10 minutes, and the clear supernatant was filtered through a 0.22 μm SCAA-104 filter (ANPEL, Shanghai, China) for subsequent LC–MS/MS analysis. Quality control (QC) samples, encompassing aliquots from all samples, were prepared to verify experimental consistency. The metabolites were quantified using a combined UPLC (Shim-pack UFLC SHIMADZU CBM30A) and MS/MS (Applied Biosystems 6500 QTRAP) system. Data processing, including filtering, peak identification, and alignment, was conducted via Analyst 1.6.1 software. Comparative group analysis utilized PLS-DA and OPLS-DA via R package models (http://www.r-project.org/), with the OPLS-DA model validated through cross-validation and a 200-permutation test. Metabolites were ranked based on their (O)PLS model VIP scores, with a VIP threshold of 1. A *t*-test (P < 0.05 and VIP ≥ 1) identified significant differential metabolites between groups.

### Statistical analysis

Data are displayed as mean ± SE, based on at least three independent experiments with three or more replicates each. Statistical assessments were conducted using the *t*-test or Tukey’s multiple test (*P* < 0.05) as suitable, employing SPSS (version 18.0).

## Acknowledgements

This research was funded by the National Natural Science Foundation of China (no. U2003113; U1803104; 31971710); China Postdoctoral Science Foundation (2505BSHJJ); A Project Funded by the Priority Academic Program Development of Jiangsu Higher Education Institutions.

## Data availability statement

The raw sequencing data that support the findings of this study have been deposited into CNGB Sequence Archive (CNSA) of China National GeneBank DataBase (CNGBdb) with project accession number CNP0005037.

## Conflict of interests

The authors declare no conflict of interest.

## Supplementary information

Supplementary data is available at *Horticulture Research* online.

## Supplementary Material

Web_Material_uhae093
