# Supplementary material for: Multi-omic dissection of the cold resistance traits of white water lily
Source: Hortic Res. 2024 Feb 17;11(6):uhae093. doi: 10.1093/hr/uhae093 (PMC11151331; doi:10.1093/hr/uhae093)
Supplement: Web_Material_uhae093 [file web_material_uhae093.zip › Supplementary Figures and Tables.docx]

## Figure S1


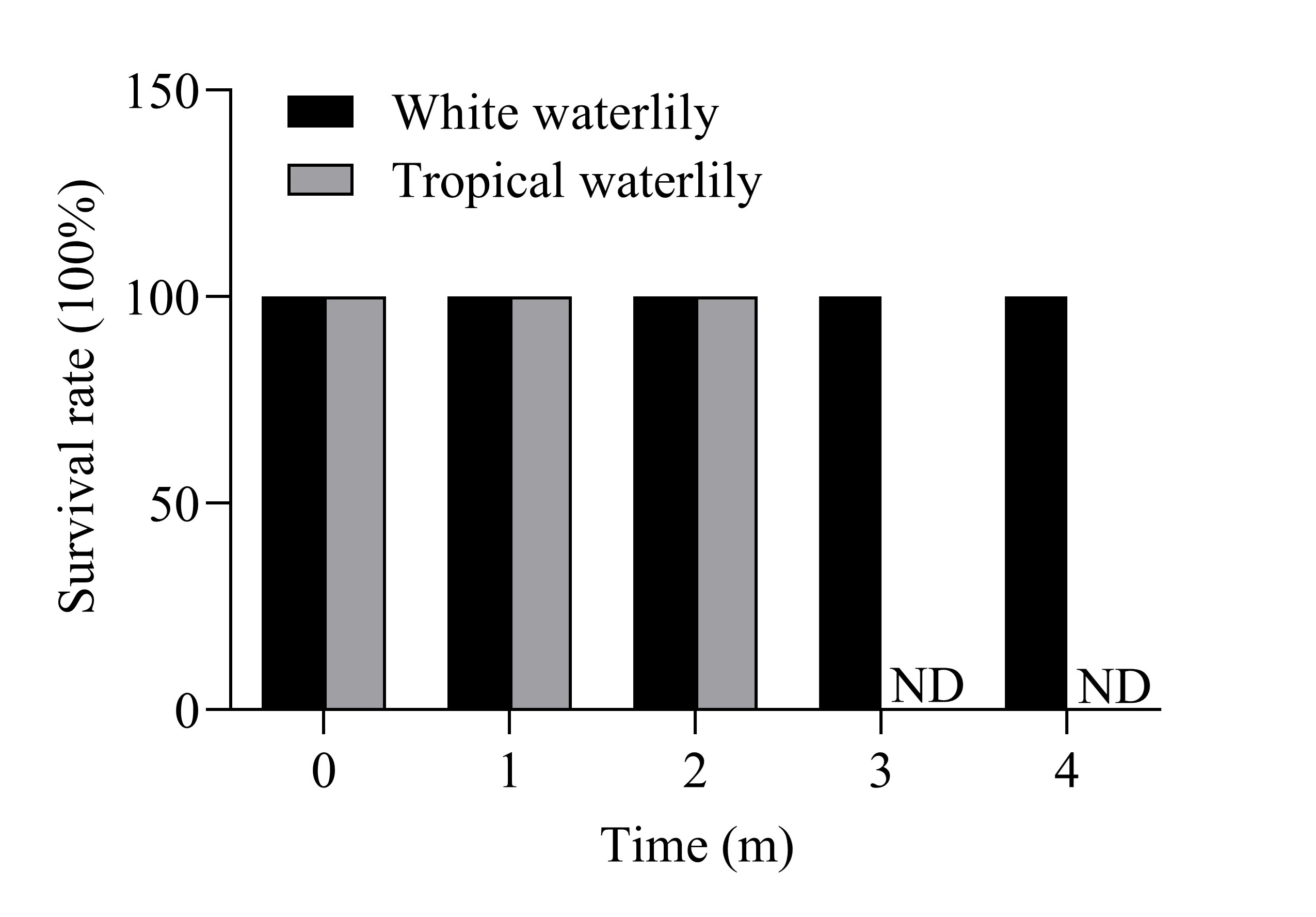


**Figure S1** Survival rates of white waterlily and tropical waterlily dormant rhizomes at 4°C. Fully dormant rhizomes of white waterlily and tropical waterlily 'Golden State' were exposed to a 4°C environment for up to 4 months. At the end of each month, the rhizomes were transferred into 25°C water to resume growth. Rhizomes that failed to germinate were considered non-viable, and the survival rates of rhizomes subjected to different durations of cold exposure were calculated. ND, not detected. The values represent means ± standard error (SE) of three independent biological replicates, with each experimental condition replicated at least three times using a minimum of five rhizomes.

## Figure S2


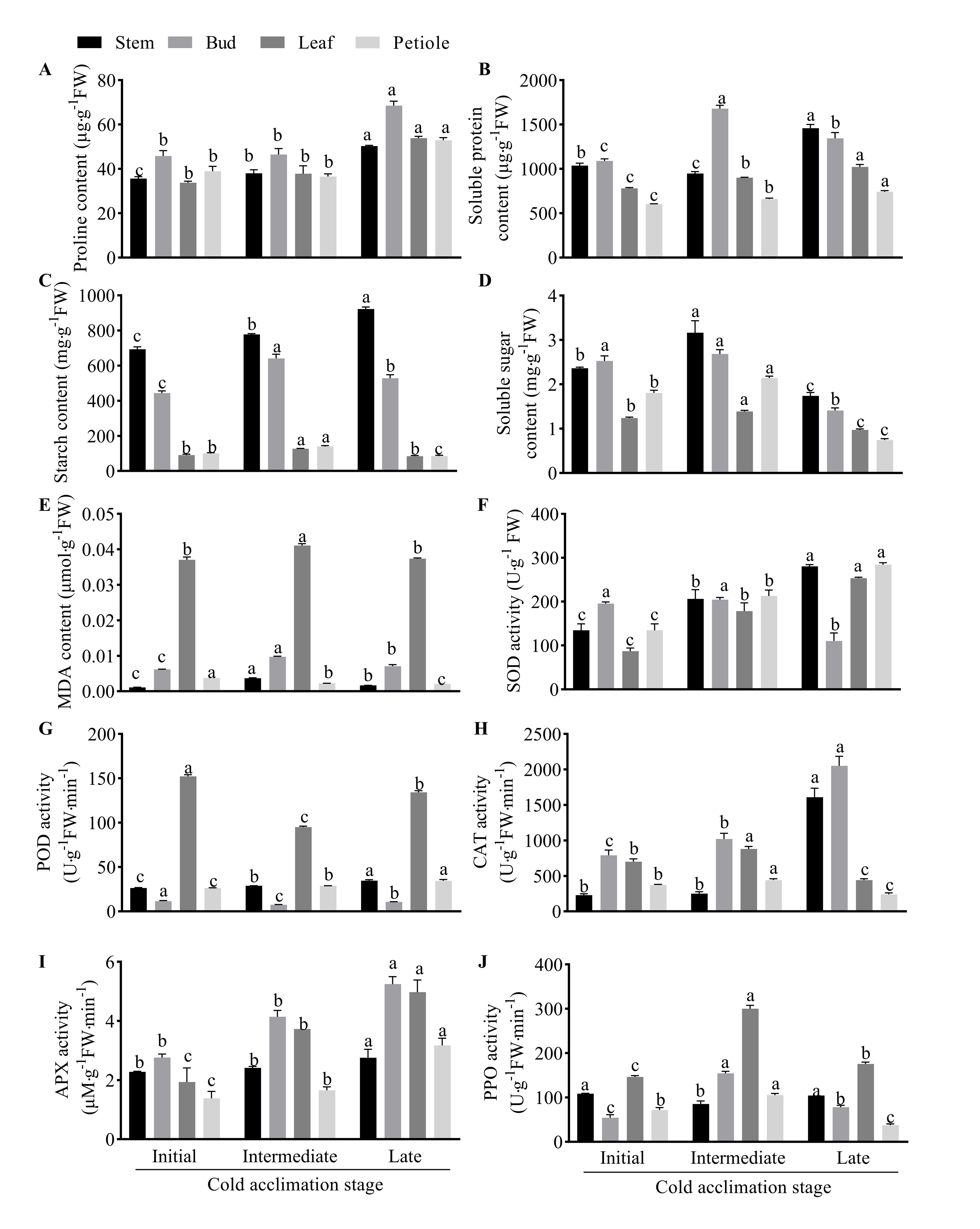


**Figure S2 Physiological changes of white water lily.** (A) Proline content; (B) Soluble protein content; (C) Starch content; (D) Soluble sugar content; (E) MDA content; (F) SOD activity; (G) POD activity; (H) CAT activity; (I) APX activity; (J) PPO activity. The value is the mean ±SE of three independent biological repeats, and each experiment is repeated at least three times. Bars with different letters are significantly different (P<0.05) according to Tukey’s multiple comparisons test.

## Figure S3


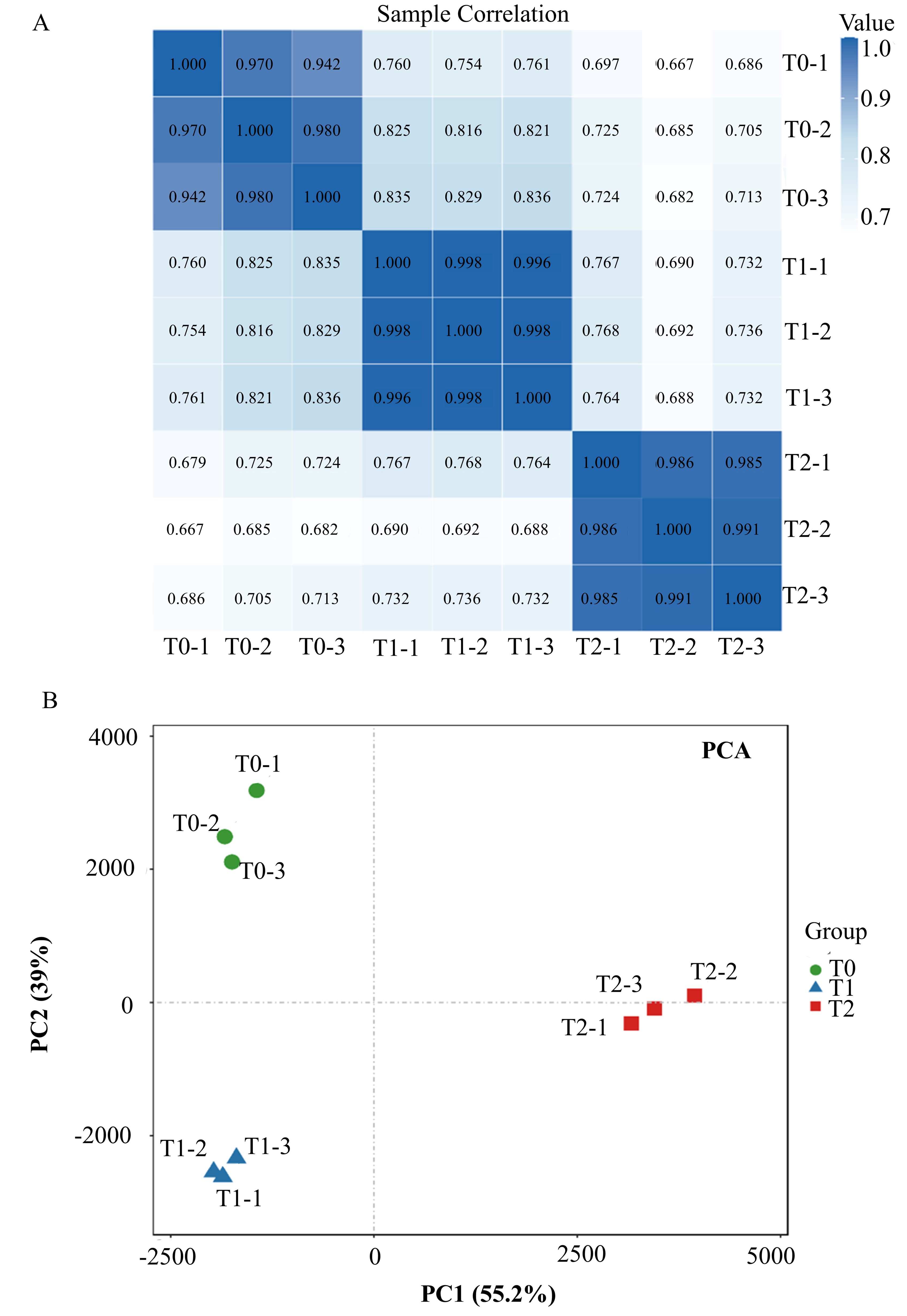


**Figure S3** **Sample correlations and principal component analysis.** (A) Heatmap of the correlations among samples. (B) PCA analysis of the samples. PC1 axis represents the first principal component with the percentage in parentheses; PC2 axis represents the second principal component, with its variance contribution indicated in parentheses. The colored dots in the plot correspond to individual samples at various stages of the study. T0, initial stage; T1, intermediate stage; T2, late stage.

## Figure S4


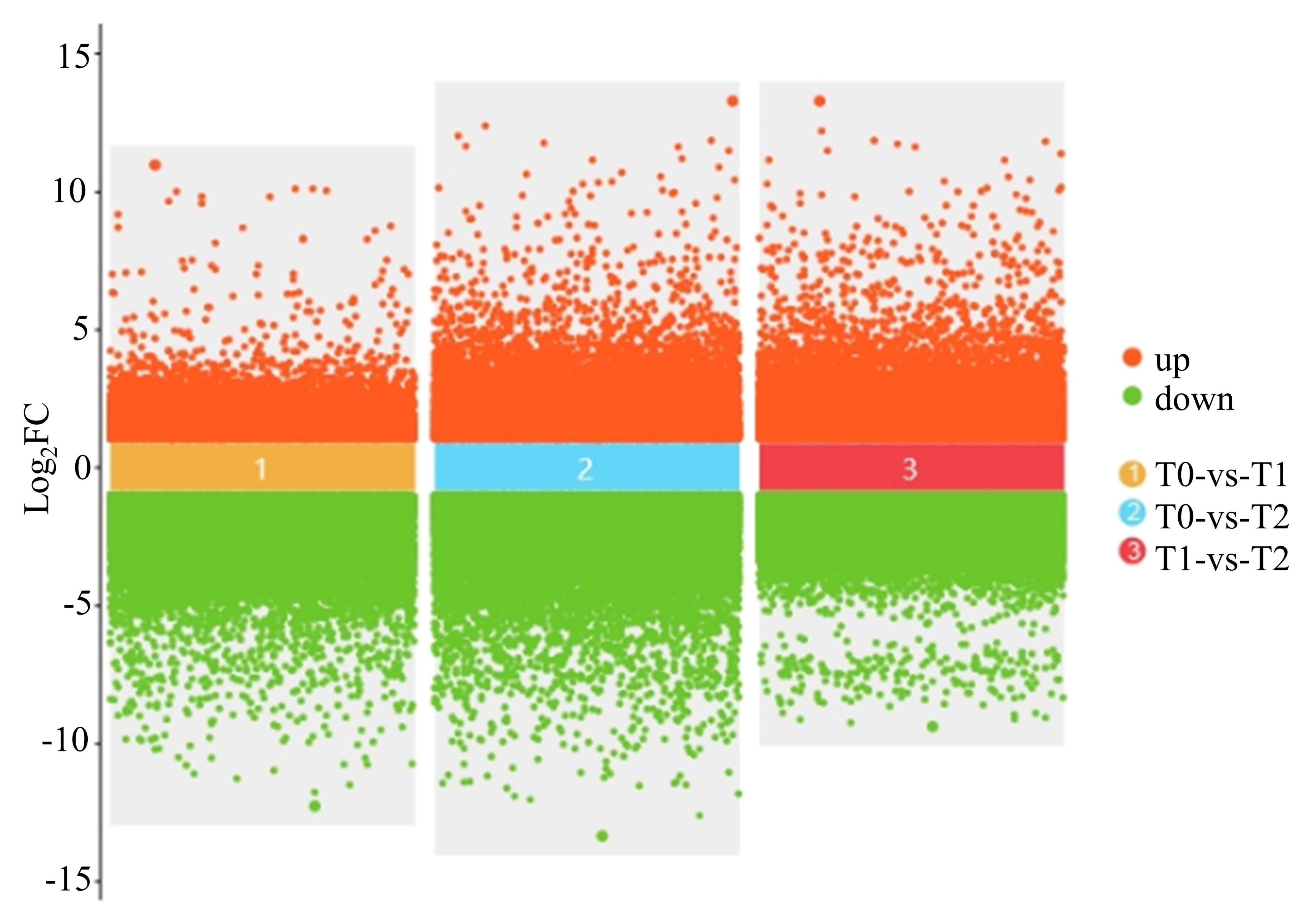


**Figure S4 Scatter plots of differentially expressed genes.** up, upregulated genes. down, downregulated genes. log_2_FC, the log2-transformed fold changes of gene expression. T0, initial stage; T1, intermediate stage; T2, late stage.

## Figure S5


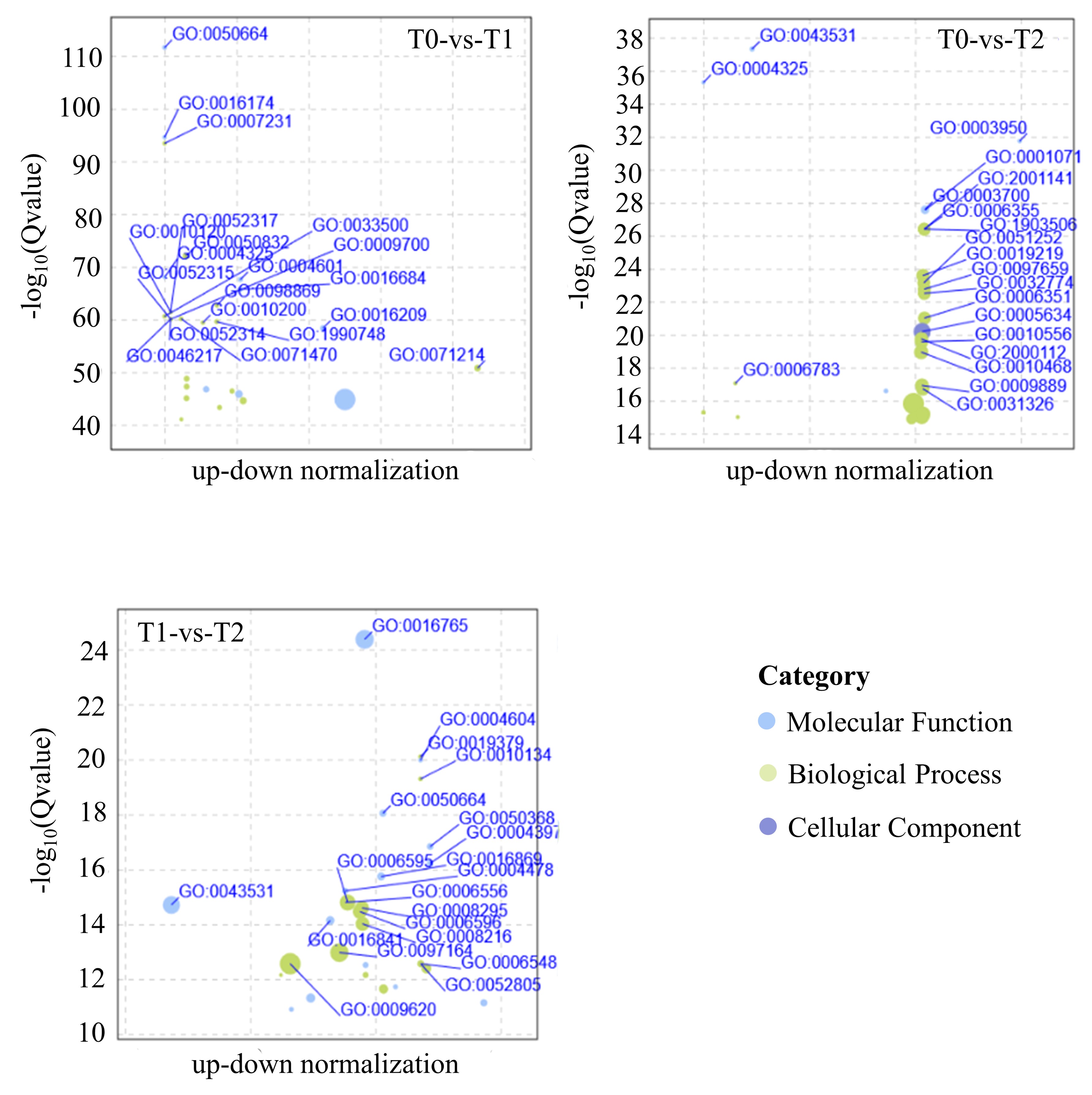


**Figure S5** **Bubble plot of top 20 significantly enriched GO terms.** The y-axis displays the -log_10_ transformed False Discovery Rate (FDR) values of the GO terms. The up-down normalization, shown on the x-axis, represents the proportion of the difference between the number of upregulated and downregulated genes relative to the total number of differentially expressed genes. The size of each bubble corresponds to the extent of enrichment of each GO term. T0, initial stage; T1, intermediate stage; T2, late stage.

## Figure S6


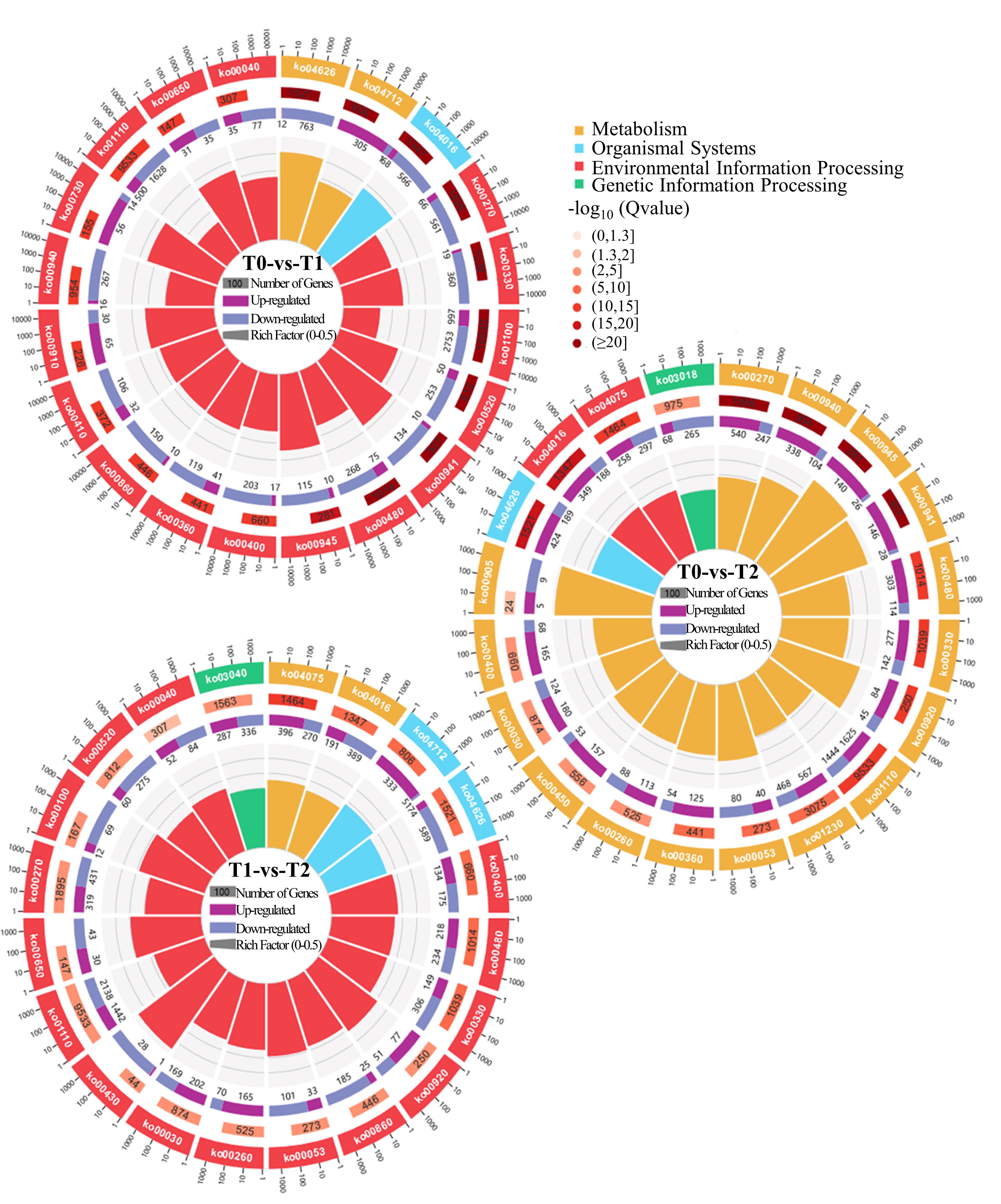


**Figure S6** **Circle plots of top 20 significantly enriched KEGG pathways.** The first circle (outermost) displays the top 20 enriched pathways, with the scale around the circle indicating the number of differentially expressed genes. Different colors represent distinct A classes of pathways. The second circle shows the number of genes in the differentially expressed gene background for each pathway and their corresponding Q values, where longer bars indicate more genes and a redder hue indicates lower Q values. The third circle provides a bar graph of the proportion of upregulated (dark purple) and downregulated (light purple) genes in each pathway, with specific numerical values displayed below. The fourth circle (innermost) illustrates the RichFactor for each pathway, calculated as the ratio of differentially expressed genes in a pathway to the total gene count in that pathway. The background grid lines in this circle represent a scale with each grid equating to 0.1. T0, initial stage; T1, intermediate stage; T2, late stage.

## Figure S7


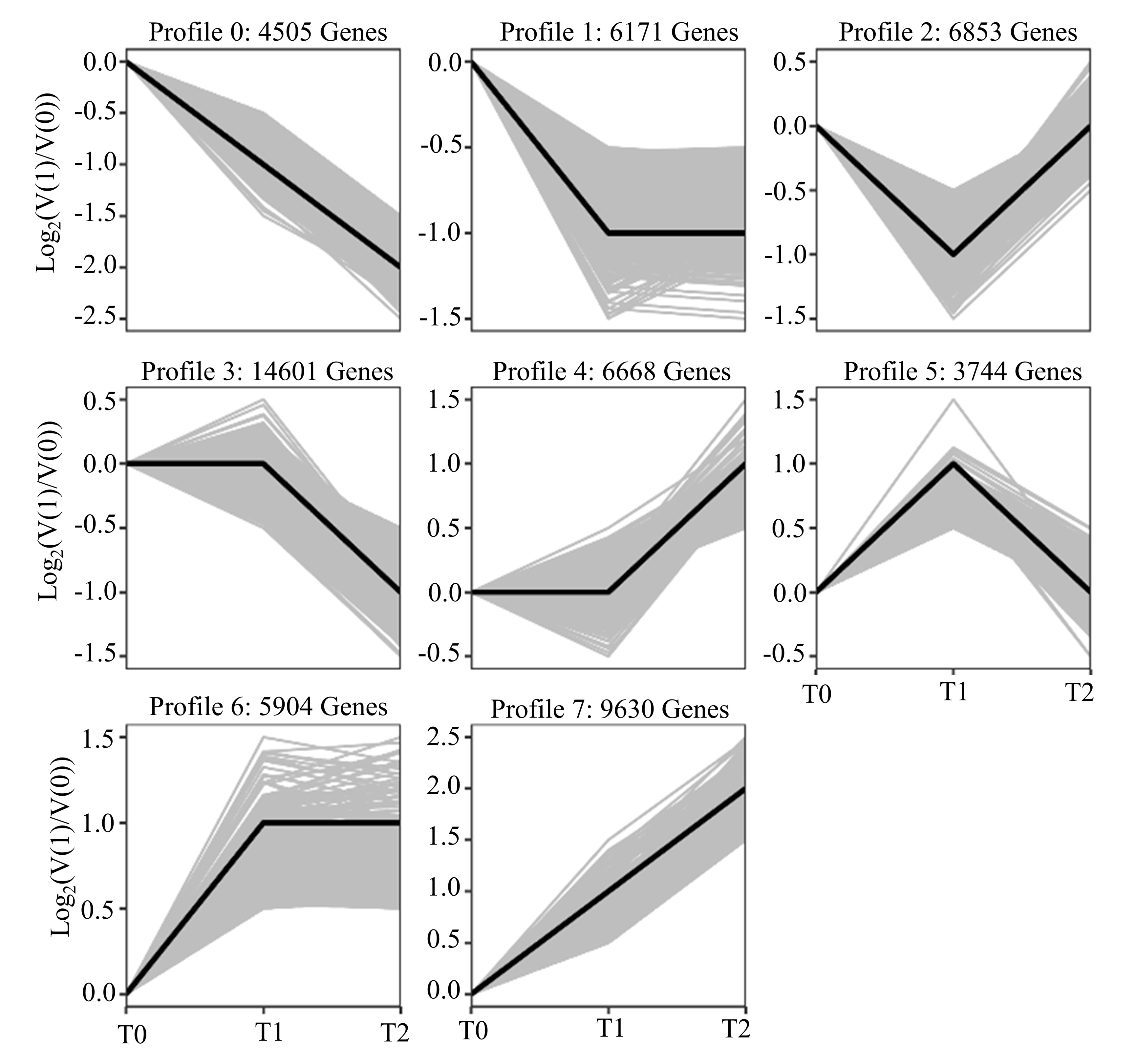


**Figure S7 Expression profiles of DEGs.** The normalized expression profiles of DEGs, categorized by their expression trends over the different cold acclimation stages T0 (initial stage), T1 (intermediate stage), and T2 (late stage). Each trend pattern is represented with individual genes displayed as gray lines, while the overarching trend for each group is highlighted with a black line.

## Figure S8


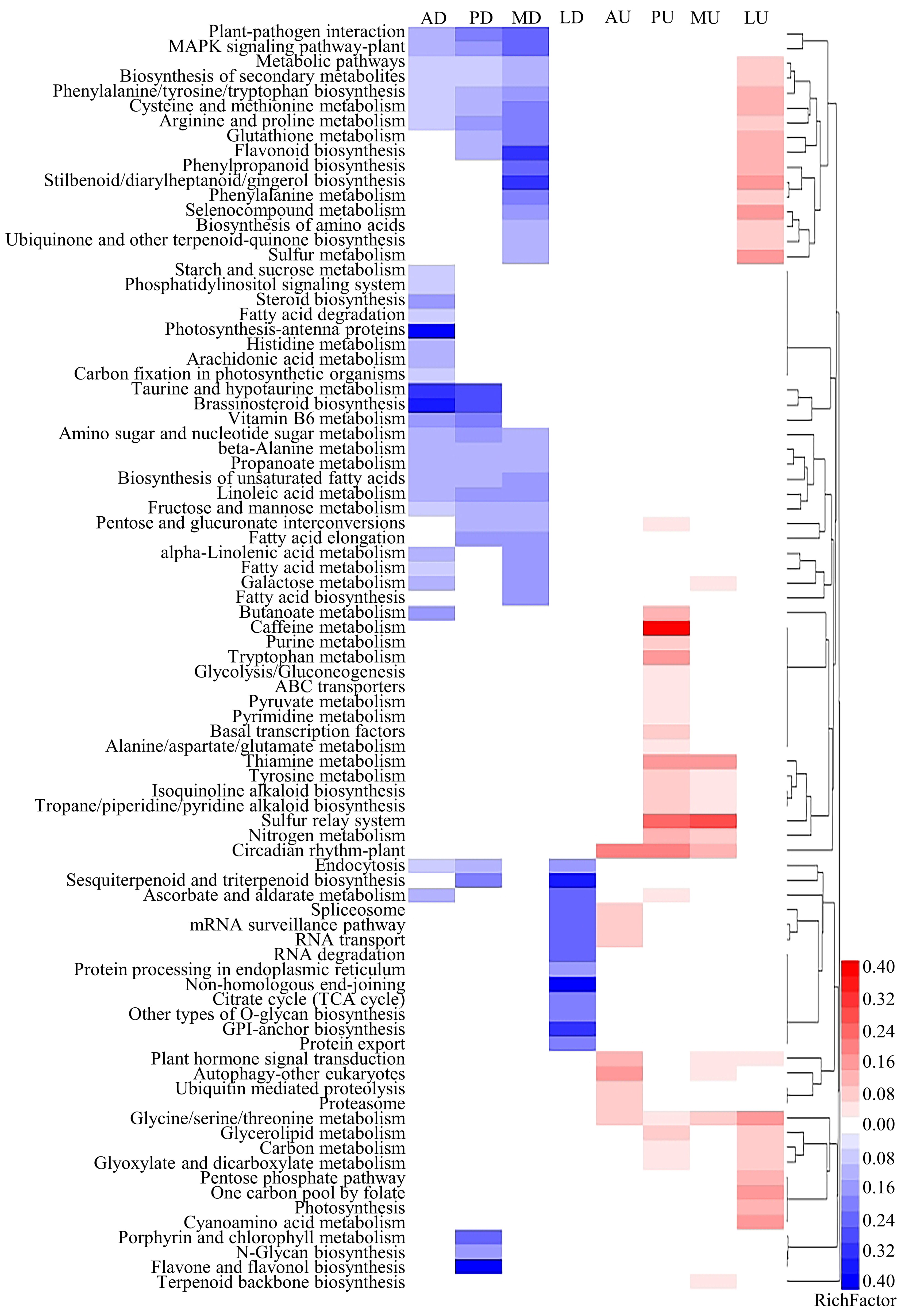


**Figure S8 KEGG analysis of genes with different expression trends.** AD, downregulation throughout the cold acclimation stage; PD, downregulation during the initial stage; MD, downregulation during the intermediate stage; LD, downregulation during the late stage; AU, upregulation throughout the cold acclimation stage; PU, upregulation during the initial stage; MU, upregulation during the intermediate stage; LU, upregulation during the late stage. The color scale represents the RichFactor values for each pathway. Upregulated genes are shown in red, while downregulated genes are shown in blue.

## Figure S9


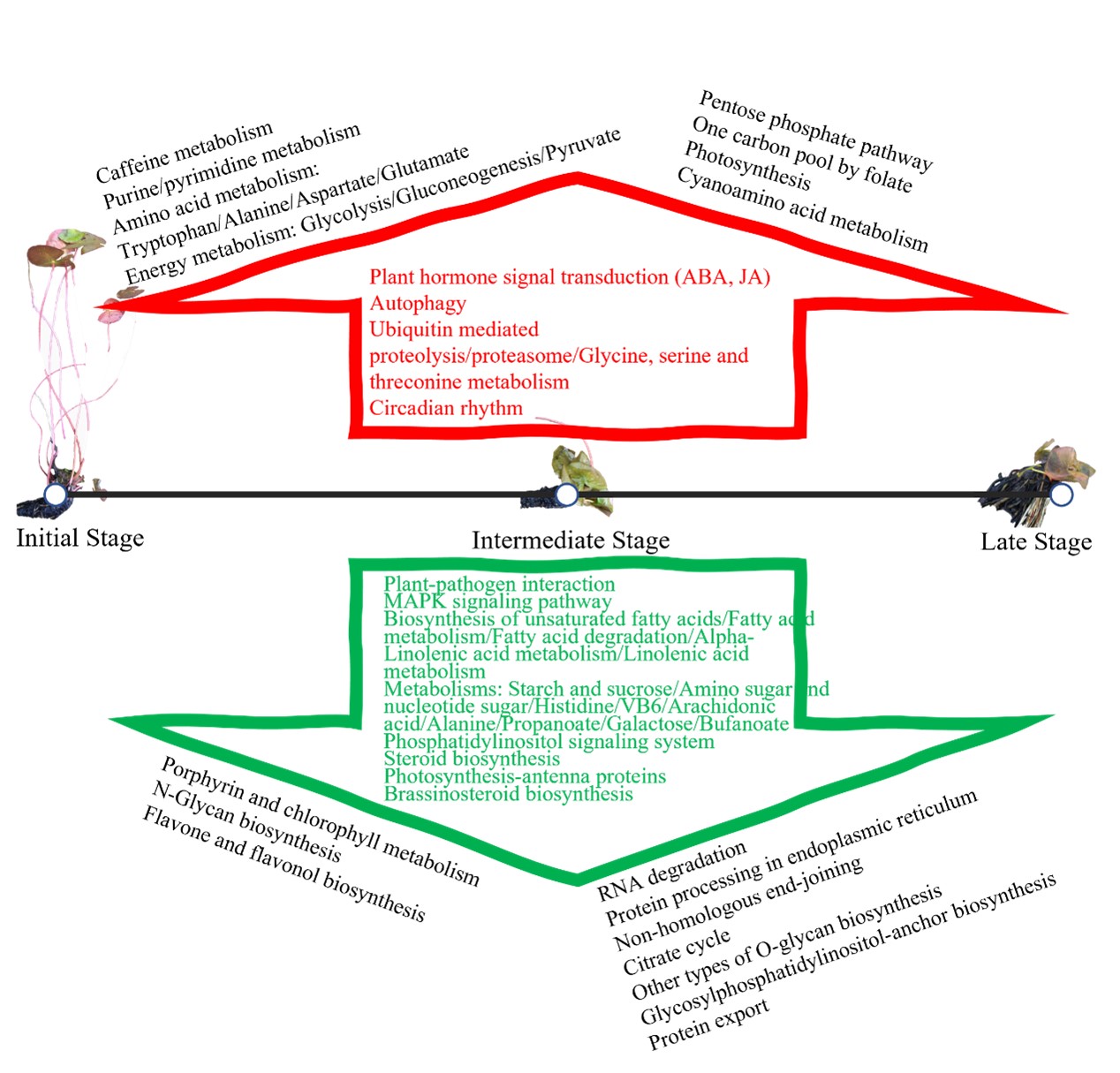


**Figure S9 Model of gene regulation during cold acclimation.**

## Figure S10


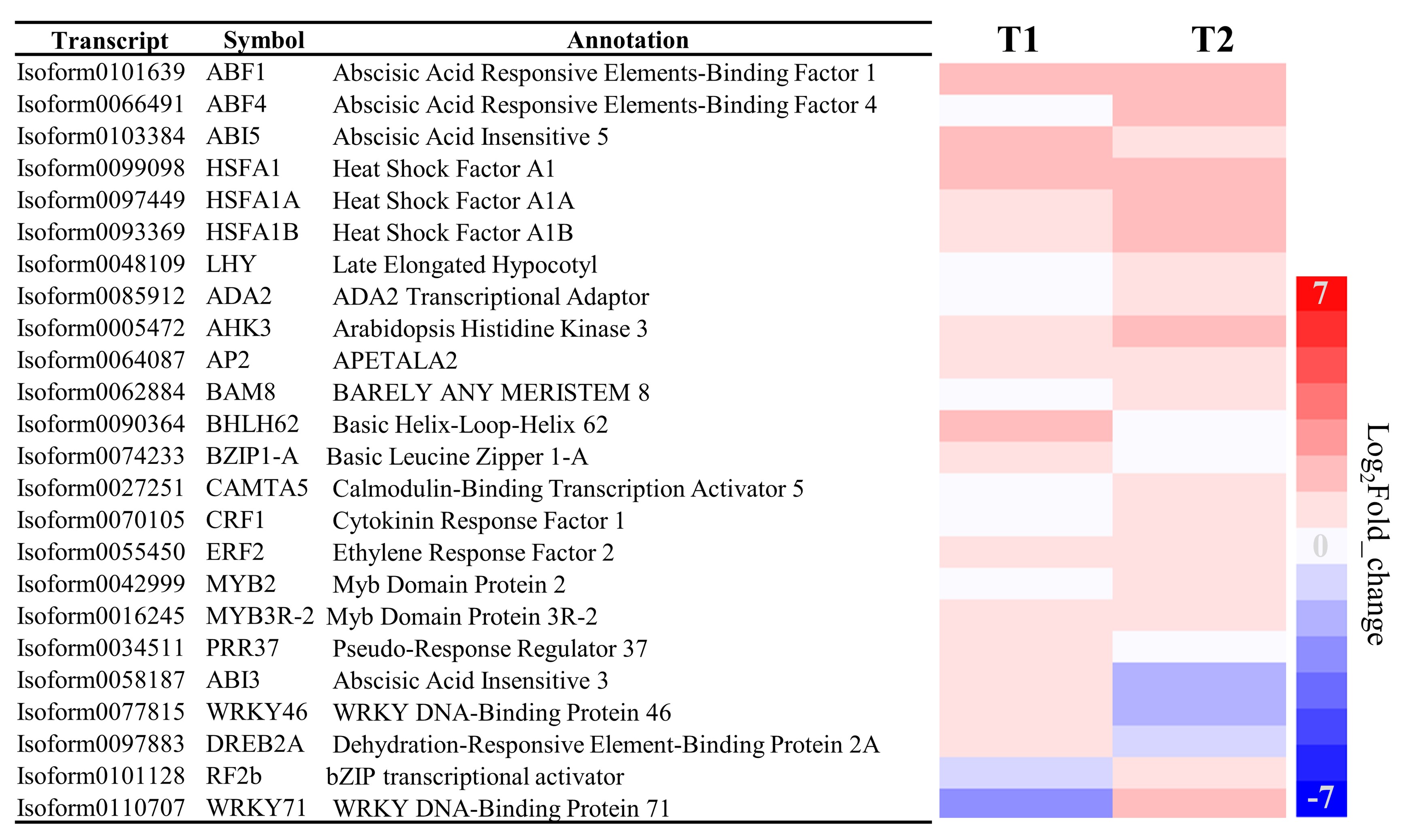


**Figure S10 Expression dynamics of DEGs encoding transcription factors during the cold acclimation process in white water lily.** A heatmap illustrates the changes in gene expression at the cold acclimation stages T1 and T2, in comparison to the T0. T0, initial stage; T1, intermediate stage; T2, late stage.

## Figure S11

**
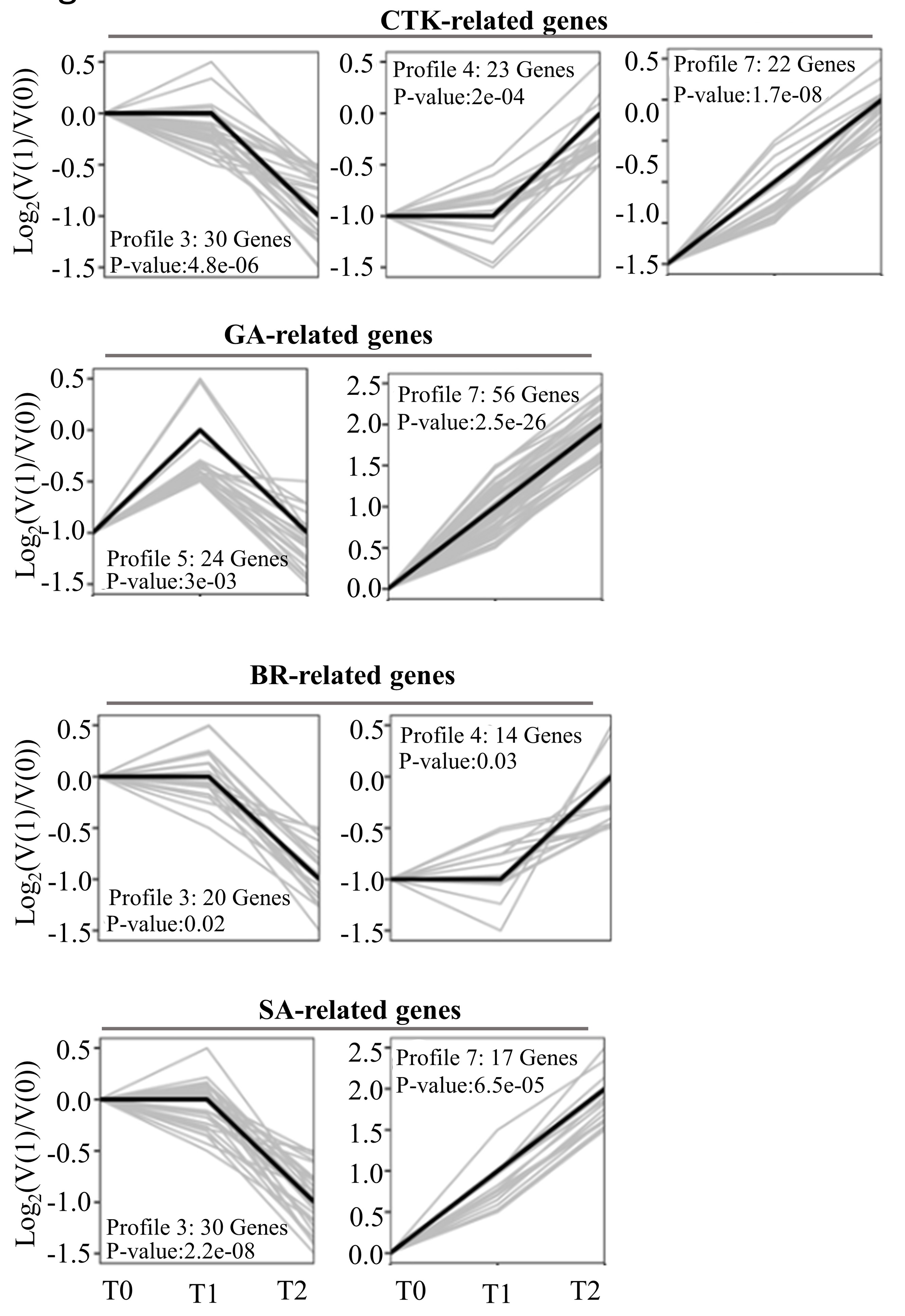
**

**Figure S11 Expression profiles of phytohormone-related DEGs.** The normalized expression profiles of phytohormone-related DEGs, categorized by their expression trends over the different cold acclimation stages T0 (initial stage), T1 (intermediate stage), and T2 (late stage). Each trend pattern is represented with individual genes displayed as gray lines, while the overarching trend for each group is highlighted with a black line.

## Figure S12

**
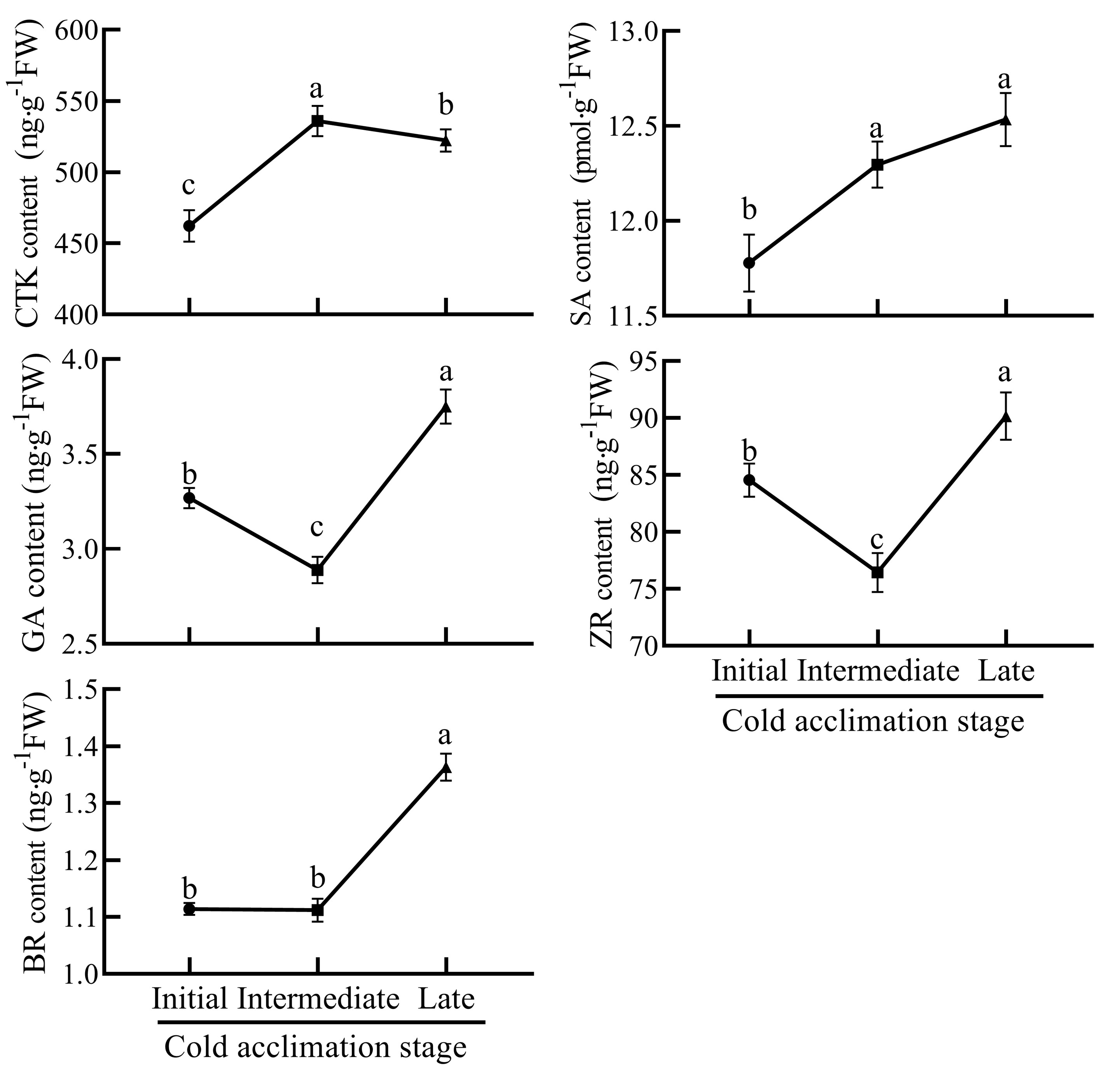
**

**Figure S12 Phytohormone content in white water lily during cold acclimation.** The value represents the mean ±SE of three independent biological repeats, and each experiment is repeated at least three times. Bars with different letters are significantly different (P<0.05) according to Tukey’s multiple comparisons test. GA, gibberellin; CTK, jasmonic acid; IAA, indole-3-acetic acid; SA, salicylic acid; BR, brassinosteroid; ZR, zeatin riboside.

## Figure S13


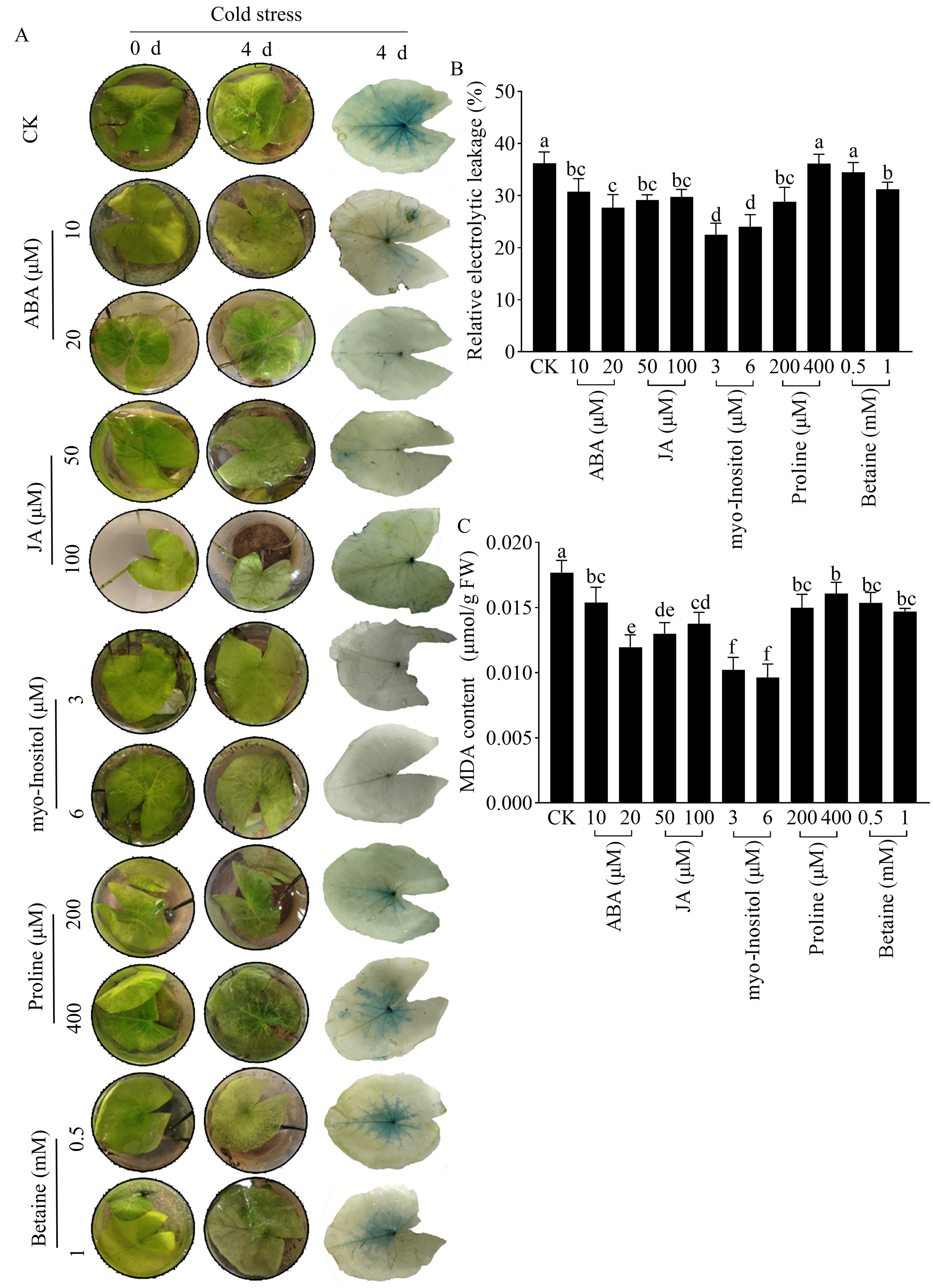


**Figure S13 Effects of exogenous chemicals on cold tolerance of the tropical water lily.** (A) Phenotypic changes in tropical water lily pretreated with different chemicals. After 4 days of cold treatment (0°C), the leaves were stained with Evans blue. (B-C) Changes of relative conductivity (B) and MDA content (C) of tropical water lily pretreated with different chemicals. The value represents the mean ± SE of three independent biological repeats, and each experiment is repeated at least three times.

## Figure S14

**
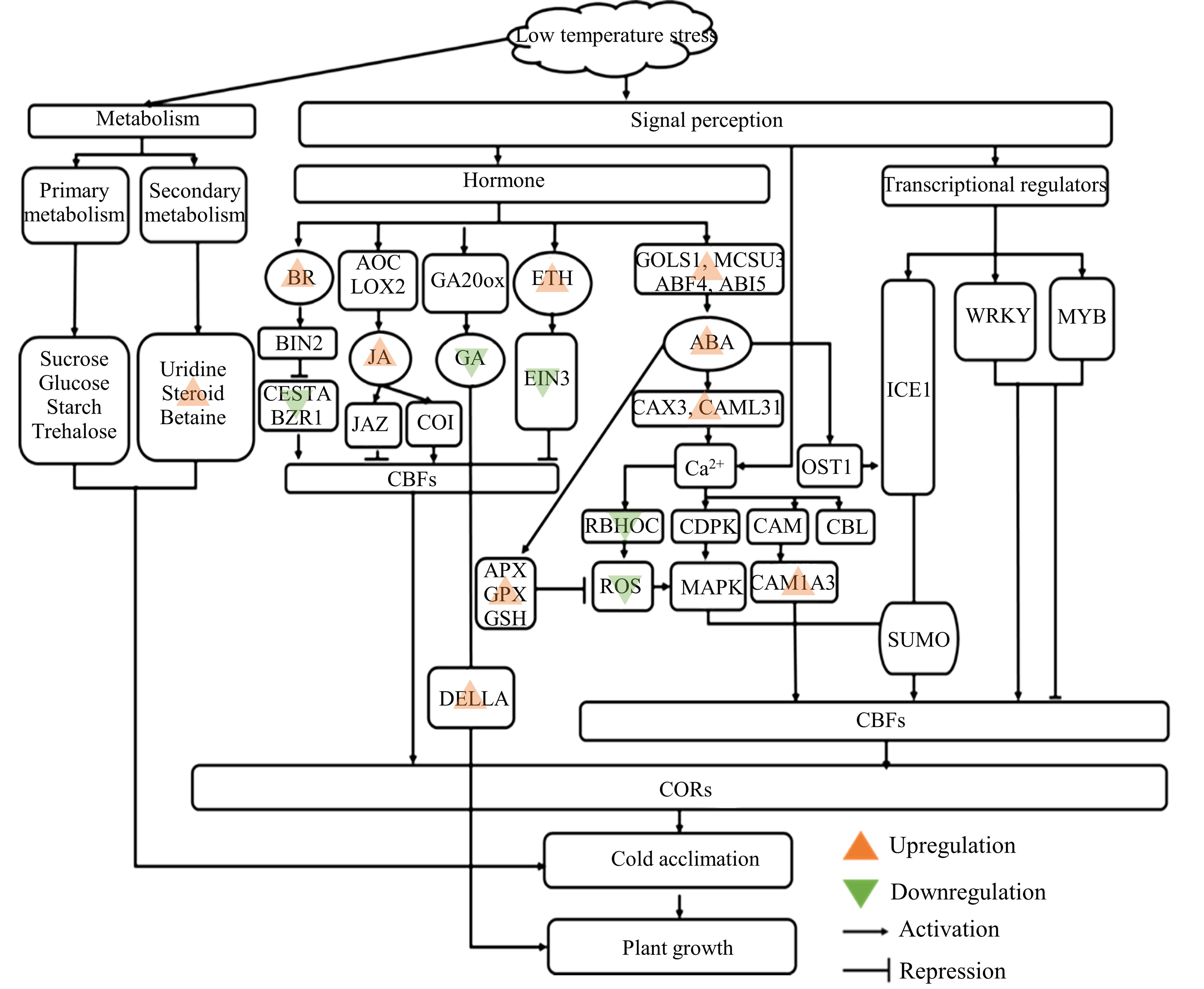
**

**Figure S14 Model of hormonal regulatory network.** AOC, Amine Oxidase; BIN2, Brassinosteroid Insensitive 2; LOX2, Lipoxygenase 2; GA20ox, Gibberellin 20-Oxidase; CAX3, Calcium/Proton Antiporter 3; CAML31, Calmodulin-Like 31; CDPK, Calcium-Dependent Protein Kinase; CAMTA, Calmodulin-Binding Transcription Activator; GSK3, Glycogen Synthase Kinase 3.

## Figure S15

**
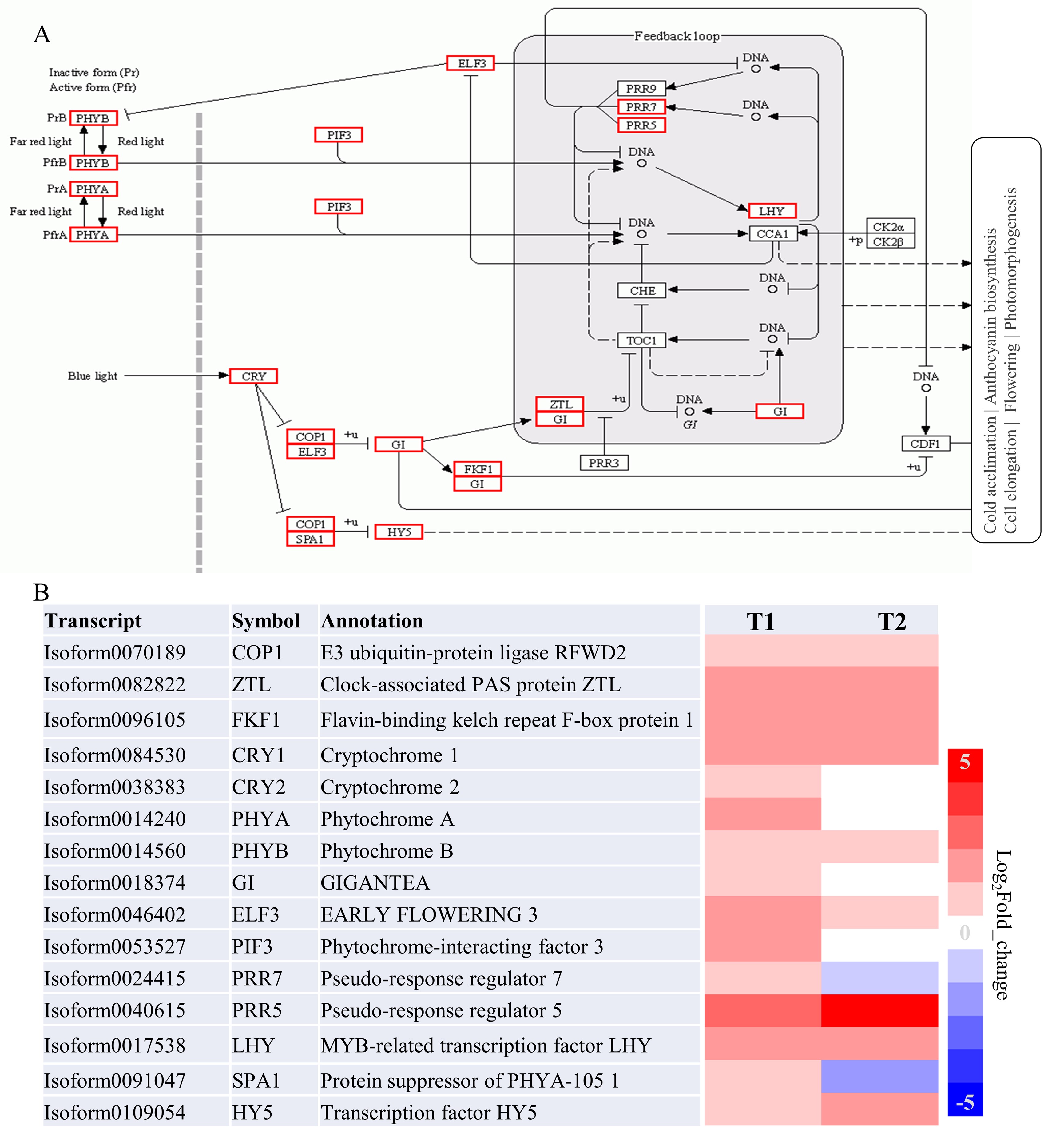
**

**Figure S15 Expression dynamics of genes within the circadian rhythm pathway during cold acclimation of white water lily.** (A) Schematic representation of the classical circadian rhythm pathway, highlighting genes with significant upregulation (denoted in red) at the T1 stage of cold exposure. (B) Heatmap of DEGs encoding genes in the circadian rhythm pathway across two time points, T1 and T2, relative to the control (T0). The color scale represents log2-transformed fold changes. T0, initial stage; T1, intermediate stage; T2, late stage.

## Figure S16


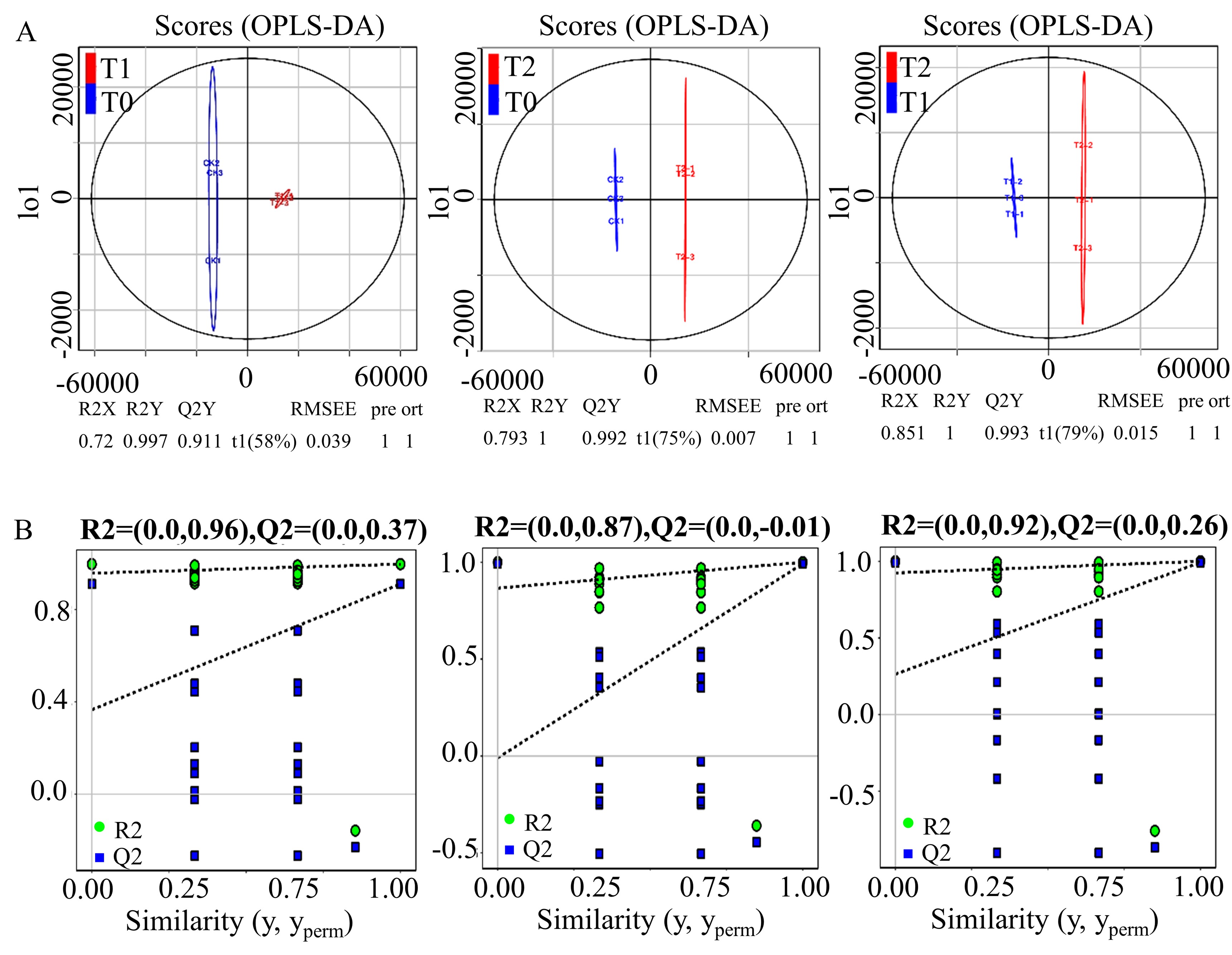


**Figure S16 Orthogonal partial least square discriminant analysis.** (A) OPLS-DA model used to differentiate groups within the data. (B) The model's effectiveness is assessed using R2X (explained variance in X), R2Y (explained variance in Y), Q2 (predictive ability), and an OPLS-DA score plot. The two points on the far right (x=1.0) represent the original model's R2 and Q2 values, while the points on the left represent the R2’ and Q2’ values from the permuted models. T0, initial stage; T1, intermediate stage; T2, late stage.

## Figure S17


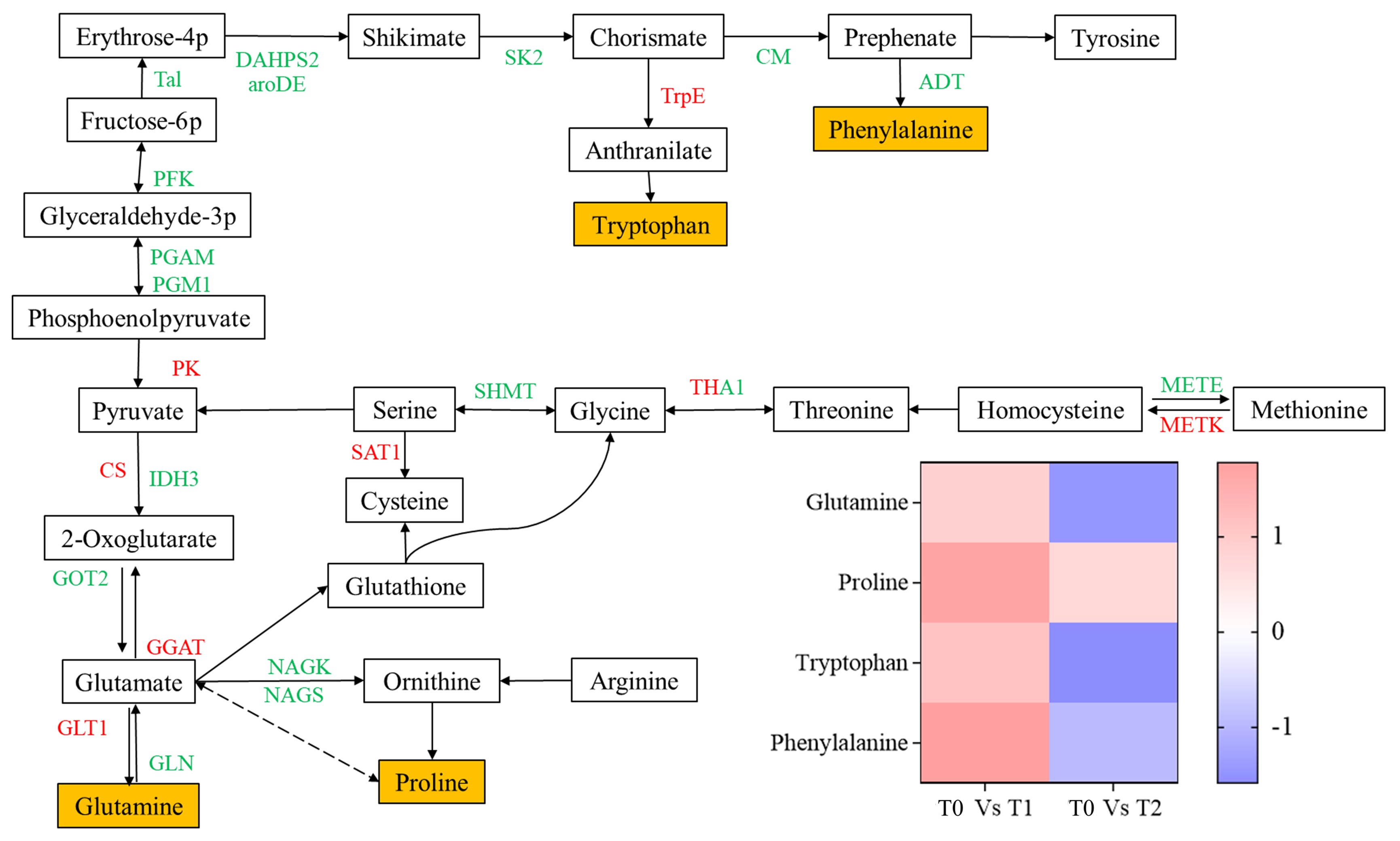


**Figure S17 Pathways of amino acid metabolism during cold acclimation.** T0, initial stage; T1, intermediate stage; T2, late stage.

## Figure S18


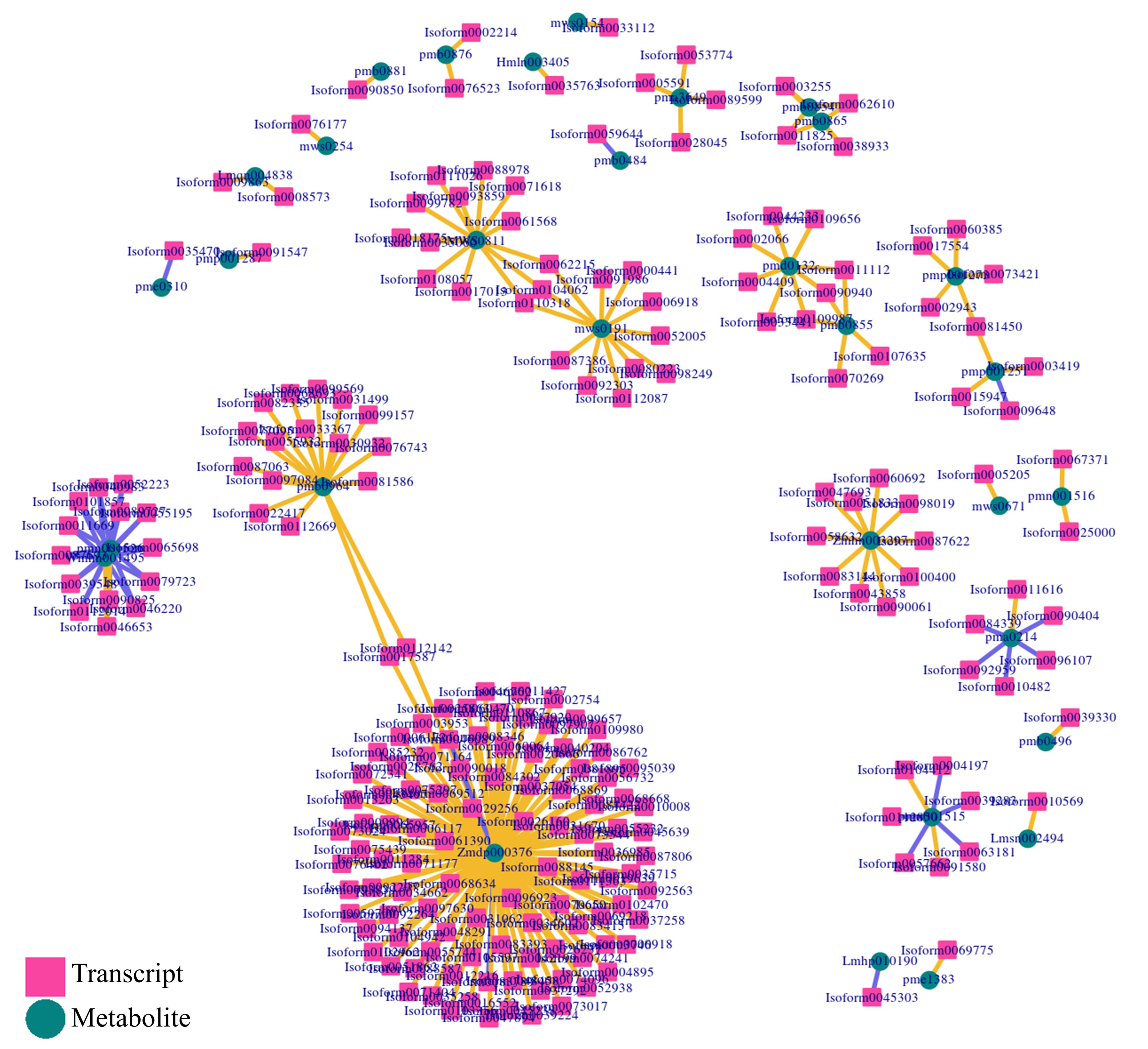


**Figure S18 The network diagram of the correlations.** Yellow lines indicate positive correlations. Blue lines denote negative correlations.

## Figure S19

**
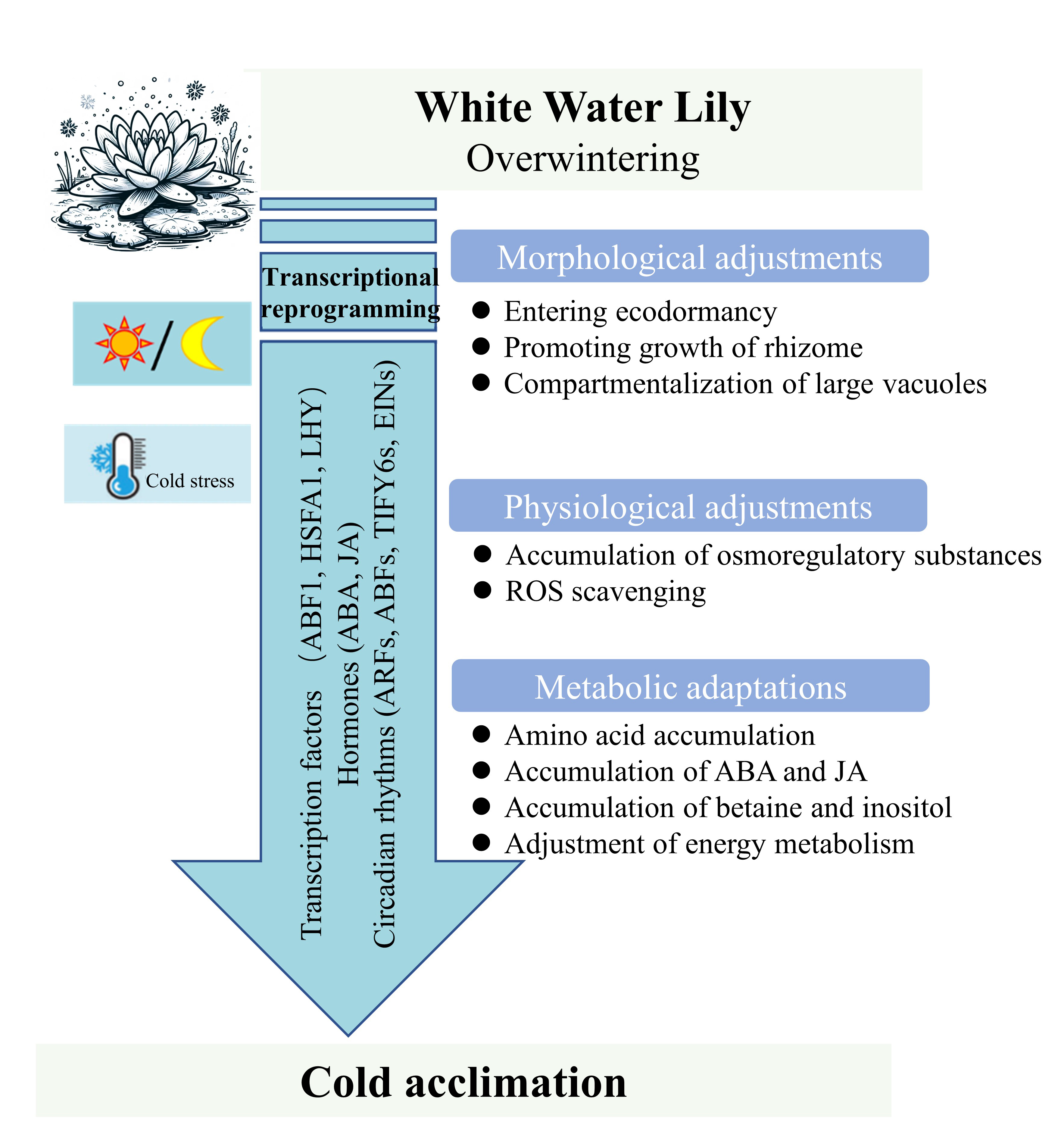
**

**Figure S19 Proposed model for the development of cold acclimation in white water lily.** In response to the persistently declining temperatures during overwintering, there are rapid changes in the levels of various transcription factors, genes related to circadian rhythms, and the content of different hormones. These changes trigger an extensive transcriptional reprogramming. This reprogramming, in turn, facilitates a comprehensive suite of morphological, physiological, and metabolic adjustments essential for cold acclimation.

| **Sample** | **RawDatas** | **CleanData(%)** | **Adapter(%)** | **LowQuality(%)** | **polyA(%)** | **N(%)** |
| --- | --- | --- | --- | --- | --- | --- |
| T0-1 | 59989996 | 59707022 (99.53%) | 24534 (0.04%) | 256636 (0.43%) | 0 (0.00%) | 1804 (0.00%) |
| T0-2 | 43510812 | 43176548 (99.23%) | 26528 (0.06%) | 306462 (0.70%) | 0 (0.00%) | 1274 (0.00%) |
| T0-3 | 46755934 | 46399932 (99.24%) | 29402 (0.06%) | 325292 (0.70%) | 0 (0.00%) | 1308 (0.00%) |
| T1-1 | 50580554 | 50327114 (99.50%) | 16264 (0.03%) | 237176 (0.47%) | 0 (0.00%) | 0 (0.00%) |
| T1-2 | 47911384 | 47654574 (99.46%) | 18148 (0.04%) | 238662 (0.50%) | 0 (0.00%) | 0 (0.00%) |
| T1-3 | 46774510 | 46543798 (99.51%) | 16446 (0.04%) | 214266 (0.46%) | 0 (0.00%) | 0 (0.00%) |
| T2-1 | 43803500 | 43563216 (99.45%) | 18712 (0.04%) | 221572 (0.51%) | 0 (0.00%) | 0 (0.00%) |
| T2-2 | 42413086 | 42087912 (99.23%) | 26832 (0.06%) | 297112 (0.70%) | 0 (0.00%) | 1230 (0.00%) |
| T2-3 | 41245824 | 40947626 (99.28%) | 23656 (0.06%) | 273404 (0.66%) | 0 (0.00%) | 1138 (0.00%) |

## Table S1 Details of the transcriptome sequencing data.

## Table S2 The composition and distribution of bases in different samples.

| **Sample** | **CleanData(bp)** | **AF_Q20(%)** | **AF_Q30(%)** | **AF_N(%)** | **AF_GC(%)** |
| --- | --- | --- | --- | --- | --- |
| T0-1 | 8923847322 | 8717359870 (97.69%) | 8330901422 (93.36%) | 143690 (0.00%) | 4249741762 (47.62%) |
| T0-2 | 6449625644 | 6286062480 (97.46%) | 6000803501 (93.04%) | 77375 (0.00%) | 3060618609 (47.45%) |
| T0-3 | 6925759893 | 6756918331 (97.56%) | 6458052185 (93.25%) | 83179 (0.00%) | 3272648483 (47.25%) |
| T1-1 | 7524060573 | 7396858134 (98.31%) | 7135314624 (94.83%) | 64952 (0.00%) | 3494746233 (46.45%) |
| T1-2 | 7114932974 | 6986228449 (98.19%) | 6725445741 (94.53%) | 62678 (0.00%) | 3300976520 (46.40%) |
| T1-3 | 6957869398 | 6833033482 (98.21%) | 6579928812 (94.57%) | 61303 (0.00%) | 3215974896 (46.22%) |
| T2-1 | 6503053555 | 6382113247 (98.14%) | 6140209669 (94.42%) | 56842 (0.00%) | 3028123021 (46.56%) |
| T2-2 | 6277552234 | 6130182156 (97.65%) | 5867862057 (93.47%) | 74658 (0.00%) | 2918156769 (46.49%) |
| T2-3 | 6112710493 | 5972951110 (97.71%) | 5722343156 (93.61%) | 73405 (0.00%) | 2831738217 (46.33%) |

## Table S8 O2PLS model parameters and prediction error.

| Model | nx | ny | n | prediction error | |
| --- | --- | --- | --- | --- | --- |
| dif | 1 | 1 | 5 | 0.857802 |  |
| T0-vs-T1 | 0 | 0 | 4 | 0.784184 |  |
| T0-vs-T2 | 0 | 0 | 4 | 0.740111 |  |
| T1-vs-T2 | 0 | 0 | 4 | 0.678011 |  |

## Table S9 The O2PLS components.

| Model | R2X | R2Y | R2Xcorr | R2Ycorr |
| --- | --- | --- | --- | --- |
| dif | 0.967 | 0.962 | 0.958 | 0.943 |
| T0-vs-T1 | 0.982 | 0.979 | 0.982 | 0.979 |
| T0-vs-T2 | 0.986 | 0.979 | 0.986 | 0.979 |
| T1-vs-T2 | 0.989 | 0.977 | 0.989 | 0.977 |
